# Beyond Thirst: Influence of Bicarbonate Mineral Water on Cardiovascular Risk Factors, Gastrointestinal Function, and Liver Health

**DOI:** 10.1002/fsn3.71183

**Published:** 2025-12-09

**Authors:** Katharina Mansouri, Maximilian Andreas Storz, Thierry Hanh, Andreas Hahn

**Affiliations:** ^1^ Institute of Food and One Health Leibniz University Hannover Hanover Germany; ^2^ Department of Internal Medicine II, Center for Complementary Medicine, Medical Center, Faculty of Medicine University of Freiburg Freiburg Germany; ^3^ Independent Researcher Paris France

**Keywords:** bicarbonate, cardiovascular, gastrointestinal, liver, mineral water

## Abstract

The role of diet in the modulation of systemic acid–base balance is of critical importance to human physiology. The contemporary dietary habits of the Western population, characterized by a high consumption of acid‐forming foods such as meat and cheese, and a low intake of vegetables and fruits, have been associated with an elevated dietary acid load. This, in turn, has been linked to a range of adverse metabolic and cardiovascular consequences. To counteract these effects, it is important to consider dietary choices. Bicarbonate‐rich mineral water offers an effective method to counteract diet‐induced acid stress, showing positive effects on cardiovascular health, gastrointestinal function, and liver metabolism. The alkalizing effect of bicarbonate‐rich mineral water can enhance insulin sensitivity through improved insulin receptor binding, resulting in an improvement in glycemic control. In terms of lipid metabolism, bicarbonate‐rich mineral water may reduce cholesterol levels by altering intestinal conditions and increasing bile acid excretion. Both, effects on glucose and lipid metabolism may positively impact cardiovascular health. Despite the high sodium content of these waters, their effect on blood pressure remains mostly neutral or positive. Benefits also extend to individuals with gastrointestinal issues such as dyspepsia and heartburn, likely due to improved gastric motility and acid‐buffering capacity. Overall, bicarbonate‐rich mineral water represents a promising nonpharmacological strategy to reduce acid load and enhance metabolic, cardiovascular, and gastrointestinal health, although further research is necessary due to variations in study designs and water composition.

## Graphical abstract

Bicarbonate‐rich mineral water may help counteract diet‐induced acid load, with potential benefits for metabolic, cardiovascular, and gastrointestinal health. It has been associated with improved insulin sensitivity, reduced cholesterol levels, and relief of dyspeptic symptoms, while its high sodium content appears to have a neutral effect on blood pressure. Despite promising findings, further research is needed due to variability in study designs and water composition.

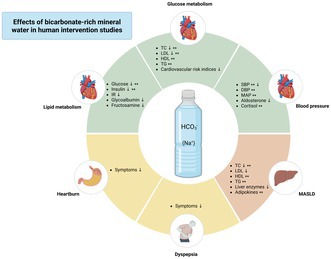

## 1 Introduction

In recent decades, as societies advance, a shift towards unhealthy eating patterns has been observed (Popkin 2017). The prevailing Western diet, characterized by a high consumption of protein derived from animals and highly processed foods combined with a low intake of vegetables and fruits, has emerged as a significant contributor to this trend (Arrieta and Aguiar 2023). Western diets result in an increased endogenous synthesis of acids, thereby inducing an acid‐based imbalance that can manifest as acid stress with low‐grade metabolic acidosis at its most severe end (Carnauba et al. 2017; Storz et al. 2022; Wesson 2021). In addition, a high dietary acid load (DAL) has been linked to a number of adverse clinical outcomes, including cardiometabolic disease and liver dysfunction (Storz et al. 2022; Wieërs et al. 2024).

These findings underline the importance of effective dietary strategies to mitigate diet‐related acid load and its subsequent health consequences. A plant‐based diet has been identified as a key strategy to ensure a high intake of base precursors. Concurrently, a reduction in sulfate‐containing proteins, which are predominantly present in animal protein, has been proposed as an effective means to reduce DAL (Adeva and Souto 2011; Siener 2011; Snelson et al. 2017; Müller et al. 2021). Additionally, minimizing highly processed foods is also beneficial given their high salt and phosphate‐based additives content (Avesani et al. 2023; Monteiro et al. 2013). Consequently, diets comprising a substantial proportion of ultra‐processed foods yield suboptimal nutritional quality, as evidenced by a diminished intake of fiber, vitamins, and minerals (Avesani et al. 2023). Furthermore, it has been observed that a high sodium chloride (salt) content has a detrimental effect on the acid–base balance, leading to hyperchloremic metabolic acidosis (Frassetto et al. 2007). In contrast, Mediterranean and Nordic diets, which are rich in fruits, vegetables, and whole grains, are potentially alkalizing. Therefore, they can reduce dietary acid load (D'Innocenzo et al. 2019; Hansen et al. 2023; Helvacı et al. 2023; Mithril et al. 2012). The same applies to vegetarian and vegan diets (Ausman et al. 2008; Kahleova et al. 2025; Müller et al. 2021; Penczynski et al. 2022; Ströhle et al. 2011).

Given the expanding body of evidence on this topic, the current paper aims to review the associations between dietary acid load and various health implications. The focus of this narrative review is on clinical outcomes related to cardiovascular, intestinal, and hepatic health. Special emphasis is directed towards the role of bicarbonate‐rich mineral water as a countermeasure, culminating in a detailed analysis of intervention studies and their potential implications for disease prevention and health promotion.

## 2 Literature Search Strategy

This narrative review is based on a comprehensive literature search. Publications up to May 2025 were screened. In the event of uncertainty, issues were discussed by the authors. Additionally, the reference lists of selected articles were screened to identify further pertinent publications. The literature search was primarily restricted to English‐language publications. However, a small number of relevant studies in French and Italian, identified through reference screening, were also included due to their relevance to the topic. The search strategy incorporated specific keywords and keyword combinations, including: “mineral water,” “water,” “bicarbonate,” “acid,” “base,” “cardiovascular,” “glucose,” “insulin,” “lipids,” “blood pressure,” “gastrointestinal tract” and “liver.” Boolean operators “AND” and “OR” were applied to refine the search results. Only full‐text articles reporting on the effects of bicarbonate‐rich mineral water were considered for inclusion. The included studies examined cardiovascular risk factors (such as glucose and lipid metabolism, and blood pressure), conditions of the upper gastrointestinal tract, as well as parameters of liver function.

## 3 Dietary Acid Load and Health Implications

A substantial body of epidemiological and clinical research has emerged, providing insights into the associations between various health implications and DAL (Wieërs et al. 2024). Within this context, the concept of Potential Renal Acid Load (PRAL) was developed by Thomas Remer and Friedrich Manz to categorize diets according to their acidic or alkaline potential (Remer and Manz 1994). Additionally, the term Net Endogenous Acid Production (NEAP) was developed. This encompasses both PRAL and the endogenously produced organic acids (OA). All of these contribute to acid excretion (Remer and Manz 1994).

DAL impacts renal function by reducing urine pH, which can lead to conditions such as hypercalciuria and stone formation (Adeva and Souto 2011; Miki et al. 2017; Trinchieri et al. 2013). Additionally, a high DAL exerts a detrimental effect on renal physiology by promoting tubular hypertrophy and glomerular hyperfiltration, which are early indicators of chronic kidney disease (CKD) (So et al. 2016; Osuna‐Padilla et al. 2019). By contrast, diets with an alkaline potential, as indicated by negative PRAL values, are associated with a higher urinary pH, as evidenced by research comparing vegan and omnivorous diets (Penczynski et al. 2022). This offers potential protective benefits against CKD and sarcopenia by decreasing glucocorticoid secretion and muscle protein breakdown (Caso and Garlick 2005; Esche 2016; Simmons et al. 1984).

In addition, results from observational studies suggest an association between DAL and several cardiometabolic risk factors. In detail, DAL may serve as an additional mechanism contributing to insulin resistance (Akter, Eguchi, et al. 2016; Moghadam et al. 2016) and impaired glucose homeostasis (Della Guardia et al. 2018), thereby increasing the risk of type 2 diabetes mellitus (Akter, Kurotani, et al. 2016; Fagherazzi et al. 2014; Kiefte‐de Jong et al. 2017; Osuna‐Padilla et al. 2019). Furthermore, a high dietary acid load is linked to high blood pressure and hypertension (Zhang et al. 2009; Krupp et al. 2018; Parohan et al. 2019; Dehghan and Abbasalizad Farhangi 2020; Lin et al. 2023; Dolati et al. 2024). The proposed underlying mechanism for both insulin resistance and hypertension involves elevated cortisol levels (Dolati et al. 2024; Maurer et al. 2003; Osuna‐Padilla et al. 2019). In addition to the well‐documented associations between DAL, insulin resistance, and hypertension, research findings have identified a potential association with hyperlipidemia (Dolati et al. 2024).

Research is still limited to the relationship between DAL and liver health, with a particular focus on metabolic dysfunction‐associated steatotic liver disease (MASLD), formerly termed nonalcoholic fatty liver disease (NAFLD) (Rinella et al. 2023). The findings suggest a positive association between increased acid load and markers of liver damage, including advanced liver fibrosis (Cheng et al. 2023; Krupp et al. 2012).

The association between high dietary acid load and osteoporosis remains debated. According to the acid‐ash hypothesis, chronic acid load may promote bone demineralization by mobilizing calcium from bone to buffer acids. However, the extent and clinical relevance of this mechanism are questioned.

Taken together, these findings highlight the complex interplay between dietary acid load and numerous health outcomes, emphasizing the potential benefits of dietary alkalization in mitigating these risks.

## 4 Mineral Water With a High Bicarbonate Content

Among the various nonpharmacological strategies for mitigating DAL, the consumption of bicarbonate‐rich mineral water has gained increasing scientific interest. This offers a physiologically relevant approach to supporting acid–base homeostasis, particularly in populations consuming Western diets.

Beyond hydration, mineral water serves as a valuable source of bioavailable electrolytes and essential ions, including calcium, magnesium, potassium, sodium, sulfate, and bicarbonate (Albertini et al. 2007; Böhmer et al. 2000; Greupner et al. 2017; Heaney 2006; Sabatier et al. 2002; Schneider et al. 2017; Ströhle and Hahn 2023), depending on its geological origin. Consequently, the consumption of highly mineralized mineral water can be considered a potential strategy for augmenting nutrient intake as a component of the daily diet (Pereira et al. 2014).

**FIGURE 1:**
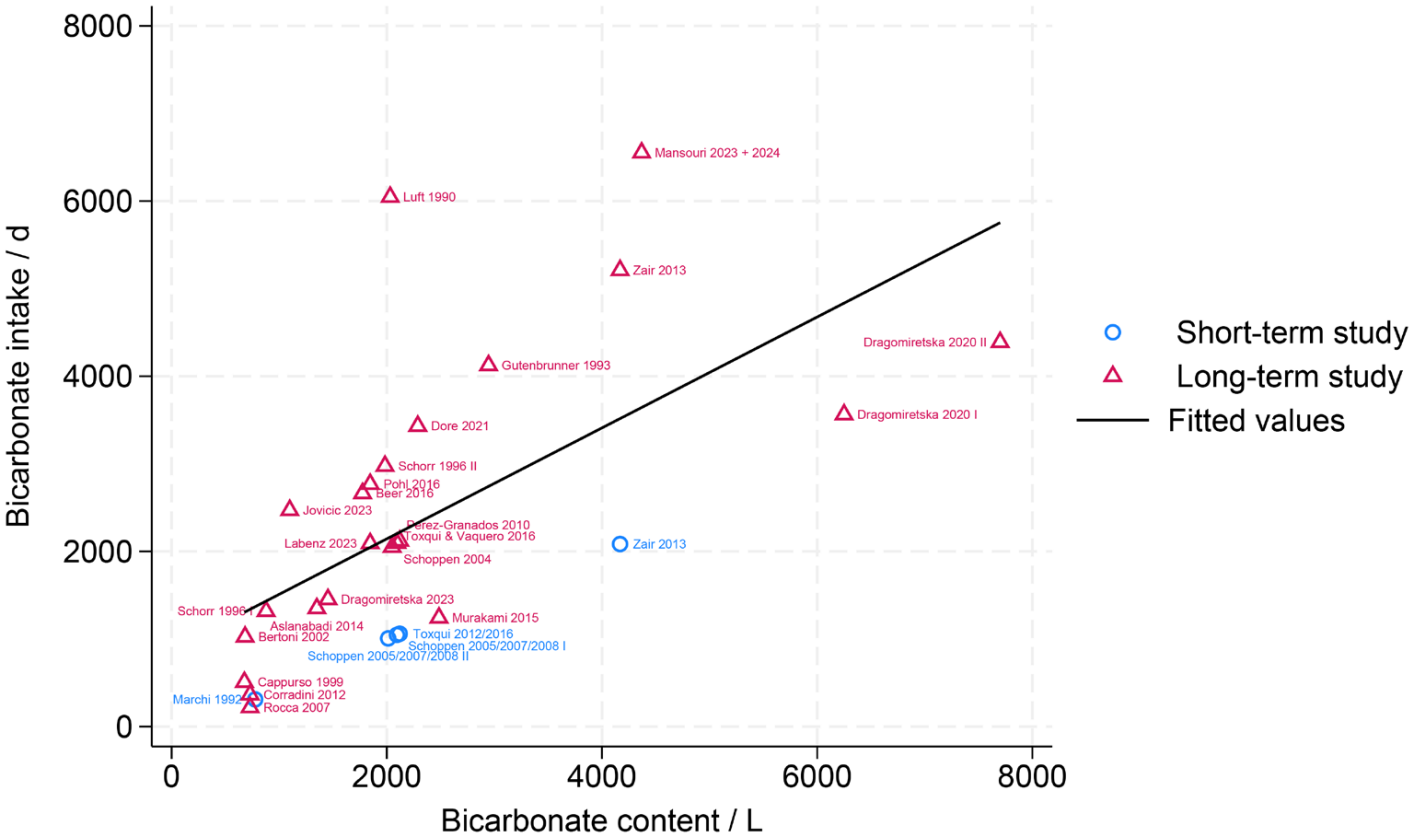
Bicarbonate amount of studied mineral waters and daily bicarbonate intake.

The legal requirements for mineral water vary by country and region. Many countries only regulate the microbiological safety and the labeling of total dissolved solids (TDS) (Food Standards Australia New Zealand FSANZ 2024; Health Canada 2025; National Health and Medical Research Council NHMRC 2025; U.S. FoodDrug Administration FDA 2009a, 2009b). The WHO only states recommendations for drinking water, including safety standards (World Health Organization 2022). There are no specific international regulations regarding the labeling of the various cations and anions present in mineral water, especially the bicarbonate content. In Europe, the European Directive established in 2009 (Directive 2009/54/EC) serves as the guiding principle. Among other things, this guideline regulates the classification of mineral water. For instance, the declaration “contains bicarbonate” is determined by the presence of a bicarbonate concentration that exceeds 600 mg per liter (Quattrini 2016). Notably, research studies contained mineral water with even higher bicarbonate contents. In these intervention studies, the mineral water utilized exhibited a bicarbonate content of at least 677 mg/L. The majority of the studies reported bicarbonate concentrations ranging from 1300 to 3000 mg/L (Figure 1). Only a limited number of studies have utilized mineral water with very high bicarbonate concentrations, with some demonstrating levels up to 7700 mg/L. As indicated in a separate publication, the mineral composition of mineral water varies depending on the geological characteristics of the source (Mansouri et al. 2025). This association extends to all mineral waters and, consequently, also to waters that contain bicarbonate. However, a closer analysis reveals that these mineral waters share certain similarities, including a higher overall mineralization level. Despite this, there are some discrepancies in the mineral composition regarding the sodium, magnesium, and calcium content. It is important to note that the discussion in this review has been limited to effects mediated by bicarbonate. However, this does not imply that other components exert no modulating effect.

Bicarbonate, when ingested, acts as a buffer, thereby neutralizing excess acids derived from various food sources (Grgic et al. 2021). In general, the health effects of bicarbonate‐rich mineral water are influenced by individual nutritional status and dietary patterns. In those with suboptimal mineral intake or acid retention, regular consumption may help restore acid–base homeostasis (Sokrateva et al. 2025). Even among individuals who maintain a balanced diet and healthy kidneys, the consumption of bicarbonate‐rich mineral water has been shown to further support physiological alkalinity, thereby reinforcing acid–base stability. Research conducted in free‐living subjects provides evidence that this type of water not only functions as a means of hydration but also enhances systemic buffering capacity (Mansouri et al. 2024; Wasserfurth et al. 2019), making it a practical and effective intervention to counteract dietary acid load. The buffering capacity of bicarbonate plays a vital role in maintaining acid–base balance and offers a compensatory mechanism in individuals with preexisting acid–base imbalances (Di Iorio et al. 2019; Kim 2021).

A growing body of research has identified a correlation between the regular consumption of bicarbonate‐rich mineral water and beneficial alterations in biomarkers associated with acid–base balance and metabolic processes. These alterations manifest as an increase in urinary pH, indicating a more alkaline environment in the urine sample (Wasserfurth et al. 2019; Mansouri et al. 2024; Marangella et al. 1996; Keßler and Hesse 2000; Siener et al. 2004; Roux et al. 2004; Schoppen, Pérez‐Granados, Carbajal, Piedra, and Vaquero 2005; Karagülle et al. 2007; Wynn, Krieg, et al. 2009; Pérez‐Granados et al. 2010; Toxqui and Vaquero 2016b; Lu et al. 2022; Chycki et al. 2021; Chiron, Thomas, et al. 2024; Chiron, Erblang, et al. 2024). This, in turn, affects the risk of kidney stones by inhibiting the crystallization and agglomeration of lithogenic substances (Gundermann et al. 2004). In addition to its role in acid–base balance, emerging research suggests that bicarbonate‐rich mineral water may confer broader health benefits. Its systemic alkalizing effects have been linked to improvements in cardiovascular risk profiles, gastrointestinal function, and liver health, as summarized in the following sections.

In the following sections, a summary of the findings from research conducted on mineral water is provided. This encompasses both acute consumption (postprandial studies) and long‐term consumption (3 days up to 8 weeks).

### 4.1 Effects of Bicarbonate‐Rich Mineral Water on Metabolic Health

#### 4.1.1 Cardiovascular Risk Factors

Cardiovascular disease (CVD) is the leading cause of death worldwide, encompassing a range of conditions affecting the heart and blood vessels (World Health Organization WHO 2024). According to the World Health Organization CVD encompasses coronary heart disease, cerebrovascular disease, peripheral vascular disease, rheumatic heart disease, congenital heart disease, and deep vein thrombosis and pulmonary embolism (WHO 2025). Several factors contribute to its development. A number of these are deemed to be nonmodifiable, such as age, sex, and genetics, while others are considered to be modifiable, including diet, smoking, obesity and diabetes (Sahın and Gunsen 2023).

**FIGURE 2:**
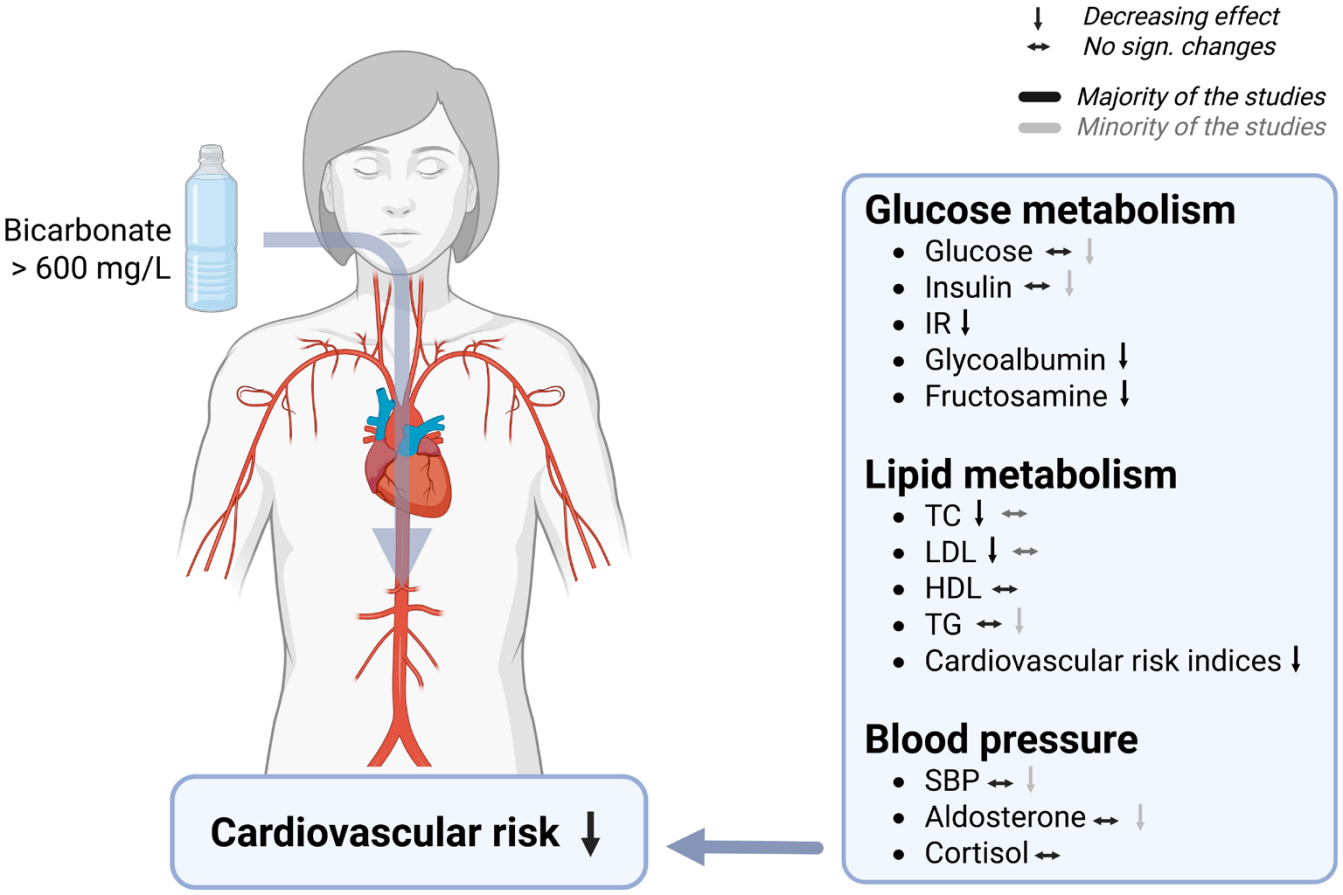
Effects of bicarbonate‐rich mineral water on cardiovascular risk factors. Created with BioRender. HDL, high‐density lipoprotein; IR, insulin resistance; LDL, low‐density lipoprotein; SBP, systolic blood pressure; TC, total cholesterol; TG, triglycerides.

Despite this, there is a paucity of studies examining the effects of dietary or supplemental bicarbonate intake on cardiovascular disease in intervention trials (Sahın and Gunsen 2023). For instance, bicarbonate supplementation in diabetic patients with chronic kidney disease has shown promising results, including improved insulin resistance, serum glucose, serum insulin, and HbA1c (Bellasi et al. 2016). Similarly, studies have reported a significant reduction in insulin resistance (assessed by HOMA‐IR) and an increase in insulin sensitivity index (assessed by the predicted insulin sensitivity index: PREDIM) among overweight individuals adhering to a vegan diet low in PRAL and NEAP (Kahleova et al. 2021). However, randomized, controlled trials investigating the chronic cardiometabolic effects of mineral water are scarce. A few intervention studies have examined the effects of acute and long‐term consumption on risk factors of cardiovascular disease, yielding promising results. However, the evidence remains inconclusive in certain aspects, necessitating further research (Figure 2).

##### 4.1.1.1 Glucose Metabolism

The effect of bicarbonate‐rich mineral water on glucose levels and related parameters of glucose homeostasis has been investigated in several randomized controlled trials, showing inconsistent results (Table 1). These studies examined the effects of mineral water consumption both in acute settings, with a single consumption of 500 mL, and long‐term settings, with a daily consumption ranging from 500 mL to 2000 mL for up to 8 weeks. Various short‐term and long‐term markers of glucose homeostasis were evaluated in individuals without impaired glucose metabolism. In the majority of the studies, the participants did not show pathological glucose levels at baseline examinations (Figure S1). No adverse effects of mineral water consumption were reported in the identified studies. However, some studies demonstrated blood glucose‐lowering effects and beneficial effects in parameters related to glucose metabolism (Dragomiretska et al. 2023; Gutenbrunner 1993; Murakami et al. 2015; Schoppen et al. 2004, 2007) while others found no significant differences compared to control water (Dore et al. 2021; Pérez‐Granados et al. 2010; Schorr et al. 1996; Zair et al. 2013). In addition, there have been studies reporting beneficial effects following the consumption of both bicarbonate‐rich mineral water and control water (Toxqui et al. 2012; Toxqui and Vaquero 2016b).

**TABLE 1:** Effects of bicarbonate‐rich mineral water on glucose metabolism.

Author	Design target group	Intervention	Characteristics of mineral water/treatment Bicarbonate/day	Main results	
				Time effects^a^ (Bicarbonate group)	Group differences time × water interaction
**Long‐term studies**					
Gutenbrunner 1993	Parallel‐group, randomized, single‐blind 24 healthy young men	4 weeks Mineral water (water A: “Überkinger Adelheidquelle”) vs. tap water (water B) 1.4 L/d	Water A: HCO_3_, Na, Mg Water B: low mineralized HCO_3_ Water A: 4124 mg/d Water B: 210 mg/d	**Fasting blood**	
				**Insulin** (serum): ↑ **Glucose:** ↔ **Fructosamine:** ↓ **OGTT** improved	(No *p*‐values reported) **Insulin**: water A ↑, water B ↔ **Glucose:** more pronounced deviations of the glucose curve in water A **Fructosamine**: water A ↓, water B ↔ **OGTT**: water A improved, water B ↔
Schorr et al. (1996)	Cross‐over, randomized, double‐blind 21 healthy older (60–72 years) individuals	NaCl reduction (< 100 mmol/d) + 4 weeks (each) 3 different mineral water brands 1.5 L/d	Water A: HCO_3_, Na, Mg Water B: HCO_3_, Na, Cl Water C: low mineralized HCO_3_ Water A: 2975 mg/d Water B: 1318 mg/d Water C: 18 mg/d	**Blood** (not specified)	
				**Insulin** (plasma): ↔ **Glucose** (plasma): ↔ **OGTT** (AUC): ↔	**Insulin** (plasma): n.s. group differences **Glucose** (plasma): n.s. group differences **OGTT** (AUC): n.s. group differences
Schoppen et al. (2004)	Cross‐over 18 postmenopausal women	2 months (each) 2 different mineral water brands 1 L/d	Water A: HCO_3_, Na, Cl Water B: low mineralized HCO_3_ Water A: 2094 mg/d Water B: 71 mg/d	**12‐h overnight fasting blood**	
				**Glucose** (serum): ↓	**Glucose** (serum): water A < water B
Pérez‐Granados et al. (2010)	Cross‐over (first water B, later water A), single‐blind 18 young hypercholesterolemic individuals	8 weeks (each) 2 different mineral water brands 1 L/d	Water A: HCO_3_, Na, Cl Water B: low mineralized HCO_3_ Water A: 2120 mg/d Water B: 104 mg/d	**12‐h fasting blood**	
				**Insulin** (serum): ↔ **Glucose** (serum): ↔ (tendency ↓ *p* = 0.056)^b^	**Insulin** (serum): n.s. group differences **Glucose** (serum): n.s. group differences
Zair et al. (2013)	Cross‐over, randomized, double‐blind 12 hypercholesterolemic men	8 weeks (each) 2 different mineral water brands (water A: “St. Yorre”; water B: “Ogeu”) 1.25 L/d	Water A: HCO_3_, Na, Cl Water B: low mineralized HCO_3_ Water A: 5210 mg/d Water B: 229 mg/d	**Fasting blood**	
				**Glucose** (plasma): ↔	**Glucose** (plasma): n.s. group differences
Murakami et al. (2015)	Cross‐over 19 healthy individuals	2 × 1 week (each) Mineral water (water A) vs. tap water (water B) 500 mL/d (before meals)	Water A: HCO_3_, Na, Ca, Mg, SO_4_ Water B: low mineralized HCO_3_ Water A: 1243 mg/d Water B: 14 mg/d	**Fasting blood**	
				Not reported	**Insulin**: n.s. group differences **Glucose** (serum): n.s. group differences (tendency ↓ *p* = 0.092)^b^ **Glycoalbumin** (serum): water A < water B **HOMA‐IR**: n.s. group differences
Toxqui and Vaquero (2016b)	Cross‐over, randomized, single‐blind 64 moderately hypercholesterolemic men and women	8 weeks (each) 2 different mineral water brands 1 L/d	Water A: HCO_3_, Na, Cl Water B: low mineralized HCO_3_ Water A: 2050 mg/d Water B: 75 mg/d	**12‐h fasting blood**	
				**Insulin** (serum): ↔ **Glucose** (serum): ↓	**Insulin** (serum): n.s. time × water interaction **Glucose** (serum): n.s. time × water interaction
Dore et al. (2021)	Single‐arm 10 patients in hospital (internal medicine)	10 days Mineral water (“San Martino”) 1–2 L/d	Water: HCO_3_, Na, Cl, SO_4_ HCO_3_ (1,5 L) Water: 3432 mg/d	**Overnight fasting blood**	
				**Glucose** (plasma): ↔	—
Dragomiretska et al. (2023)	Parallel‐group, randomized 71 men and women with chronic viral hepatitis C (concomitant nonalcoholic fatty liver disease) + after COVID‐19 diagnosis	2 months Diet + exercise (control) vs. diet + exercise + mineral water (“Shayanskaya”) 3 mL water/kg body weight, before and after each meal (3 meals)	Water: HCO_3_, Na, Si HCO_3_ Individualized (1453 g/L)	**Blood** (not specified)	
				**Glucose** (serum): ↓ **Insulin**: ↓ **HOMA‐IR‐Index**: ↓	**Glucose** (serum): n.s. group differences **Insulin**: water < control **HOMA‐IR‐Index**: water < control
Jovicic et al. (2023)	Single‐arm 60 individuals with diagnosed type 2 diabetes mellitus	28 days Mineral water (“Sneznik‐1”) 1.5 to 3 L (depending on disease stage and general condition)	Water: HCO_3_ HCO_3_ individualized	**Blood (not specified)**	
				**Glucose** (plasma): ↔	—
**Acute studies**					
Schoppen et al. (2007)	Cross‐over, randomized 18 postmenopausal women	— 3 different mineral water brands Standardized meal (fat‐rich) 500 mL	Water A: HCO_3_, Na (higher than water B), Cl Water B: HCO_3_, Na, Cl Water C: low mineralized HCO_3_ Water A: 1047 mg Water B: 1007 mg Water C: 36 mg	**12‐h overnight fasting blood + postprandial**	
				**Insulin**: ↓ **Glucose**: ↔ Peak concentration/time to peak: Not reported	**Insulin** (at 120 min): sign. time × water interaction (water B < water C) **Glucose**: n.s. group differences Peak concentration/time to peak: **Insulin**: n.s. group differences (peak, time to peak) **Glucose**: n.s. group differences (peak, time to peak)
Toxqui et al. (2012)	4 way cross‐over, randomized 21 young (> 18–< 40 years) moderately hypercholesterolemic individuals	— 2 different mineral water brands Standardized meal (fat‐rich)/without meal 500 mL	Water A: HCO_3_, Na, Cl Water B: low mineralized HCO_3_ Water A: 1060 mg Water B: 52 mg	**12‐h overnight fasting blood** (+ postprandial)	
				**Without meal** **Insulin** (serum): ↔ **Glucose** (serum): ↔ **With meal** **Insulin** (serum): ↓ **Glucose** (serum): ↔	**Without meal** **Insulin** (serum): n.s. group differences **Glucose** (serum): n.s. group differences **With meal** **Insulin** (serum): n.s. group differences (tendency: water A < water B)^b^ **Glucose** (serum): n.s. group differences
Zair et al. (2013)	Cross‐over, randomized, double‐blind 12 hypercholesterolemic men	— Two separate postprandial cycles (before + after 8 weeks treatment) 2 different mineral water brands (water A: “St. Yorre”; water B: “Ogeu”) Standardized meal 500 mL	Water A: HCO_3_, Na, Cl Water B: low mineralized HCO_3_ Water A: 2084 mg Water B: 92 mg	**Fasting blood**	
				Area under the curve (AUC) **Glucose:** ↔ between two time points	Area under the curve (AUC) **Glucose**: n.s. group differences

*Note:* Minerals: Ca, calcium; Cl, chloride; HCO_3_, bicarbonate; Mg, magnesium; Na, sodium; SO_4_, sulfate. Blood parameters: HOMA‐IR, homeostasis model assessment—insulin resistance; OGTT, oral glucose tolerance test. ↑ = significant increase (*p* < 0.05); ↔ = no significant change (*p* > 0.05); ↓ = significant decrease (*p* < 0.05).

^a^ Between begin and end of each intervention period.

^b^ Categorization of the authors.

In studies showing no significant effect on **glucose** metabolism, neither fasting glucose nor fasting insulin levels were affected by long‐term water consumption (Dore et al. 2021; Jovicic et al. 2023; Murakami et al. 2015; Pérez‐Granados et al. 2010; Schorr et al. 1996; Zair et al. 2013). Two of these studies reported a tendency towards lower glucose levels after the bicarbonate‐rich water period (Murakami et al. 2015; Pérez‐Granados et al. 2010). However, reductions in glucose levels of 2 mg/dL (Murakami et al. 2015) and 3.6 mg/L (Pérez‐Granados et al. 2010) are not regarded as clinically relevant. In contrast, a reduction in serum glucose levels has been demonstrated in healthy postmenopausal women and moderately hypercholesterolemic young to mid‐age individuals, following a daily consumption of 1000 mL of bicarbonate‐ and sodium‐rich mineral water (Schoppen et al. 2004; Toxqui and Vaquero 2016b). In postmenopausal women, the consumption of bicarbonate‐rich mineral water led to 6.7% lower glucose levels compared to the consumption of low mineralized mineral water (Schoppen et al. 2004). In individuals with diagnosed hypercholesterolemia, the decrease in glucose levels within the bicarbonate group was minimal, with a reduction of approximately 3.0% (Toxqui and Vaquero 2016b). Yet, in the study with hypercholesterolemic individuals, the reduction in glucose was possibly due to changes in beverage choice, leading to reduced carbohydrate and energy intake, rather than solely attributable to mineral water consumption during the intervention phase. Moreover, glucose levels decreased in both intervention groups, resulting in nonsignificant group differences (Toxqui and Vaquero 2016b). In the context of these studies, it is noteworthy that the participants did not have elevated glucose levels or a diagnosis of diabetes mellitus at the baseline examinations. Consequently, a clinically relevant decrease in blood glucose levels was not anticipated. However, even in individuals diagnosed with type 2 diabetes mellitus, no significant reduction in glucose levels was reported (Jovicic et al. 2023). Intake of glucose‐lowering medication might be the reason for nonfindings in individuals with diabetes. However, the absence of data regarding medication use in this study complicates the interpretation of the data. In contrast, a recent Ukrainian study demonstrated beneficial effects on glucose metabolism in individuals with impaired glucose tolerance (Dragomiretska et al. 2023). At the beginning of the study, the subjects exhibited slightly elevated glucose and insulin levels, which were marginally above the normal reference range. Following the consumption of mineral water, a decline in glucose levels was observed. Although this reduction reached statistical significance only in the mineral water group, but not the control group, no significant between‐group differences were observed. In contrast, **insulin** levels were positively affected in this study. While insulin levels decreased in the mineral water group, no significant changes occurred in the control group. As a consequence of the changes in glucose and insulin levels, the HOMA‐IR index showed significant group differences with a reduction in the mineral water group (Dragomiretska et al. 2023). Similar findings were reported in an acute study comparing the consumption of 500 mL of bicarbonate and sodium‐rich mineral water with a low‐mineralized mineral water alongside a high‐fat meal. Serum insulin levels were reduced in both study groups, with a trend towards lower levels in the bicarbonate group (Toxqui et al. 2012). However, no exact *p*‐values for group differences were reported. This finding aligns with another acute study utilizing similar amounts of slightly different bicarbonate‐rich mineral waters, which demonstrated a significant reduction in insulin levels 120 min after meal consumption, suggesting an improvement in insulin sensitivity, particularly notable in individuals with a higher HOMA index (Schoppen et al. 2007). Glucose levels were not affected in acute consumption studies (Schoppen et al. 2007; Toxqui et al. 2012; Zair et al. 2013).

Beyond short‐term benefits, two studies focused on long‐term glycemic control markers. In these studies beneficial effects were reported (Gutenbrunner 1993; Murakami et al. 2015). In one study, the consumption of 1400 mL per day of bicarbonate‐ and magnesium‐rich mineral water for a duration of 4 weeks resulted in reduced fructosamine levels and improved oral glucose tolerance (OGTT) in young healthy male individuals. In contrast, there were no significant changes in the control group (Gutenbrunner 1993). In line with these results, glycoalbumin levels were significantly lower in individuals consuming bicarbonate‐rich mineral water compared to low mineralized mineral water in a trial conducted over 2 weeks, with intervals separated by a week of tap water consumption (Murakami et al. 2015).

The disparity between short‐term and long‐term parameters is an issue that should be taken into consideration. However, it is widely acknowledged that a single measurement of fasting glucose level is not sufficient for diagnostic purposes due to its significant variability (Sacks 2011; Selvin et al. 2007). Consequently, single measurements are not regarded as reliable parameters. For this reason, it is imperative to rely on long‐term parameters when assessing the metabolic status, particularly in cases of glucose metabolism disorders, as these parameters offer a comprehensive reflection of the long‐term metabolic status by glycosylation (Nathan 2009). Given the fact that long‐term parameters have shown favorable changes, it can be posited that bicarbonate‐rich mineral water may exert a beneficial effect on glucose metabolism.

###### Mechanisms

DAL leads to a reduction in glucose uptake by the muscles and inhibition of the insulin signaling pathway (Carnauba et al. 2017). Therefore, lowering the impact of a high acid load by mineral water consumption may affect insulin resistance.

The improvement of glycemic control and reduction of insulin resistance may be due to increased insulin receptor sensitivity resulting from the alkaline nature of the mineral water (Gutenbrunner 1993). This is in line with the postulated link between a higher DAL and a disruption of insulin binding to the insulin receptor (Williams, Kozan, and Samocha‐Bonet 2016). Supporting evidence is provided by a cell culture study demonstrating impaired insulin binding following extracellular pH reduction (Hayata et al. 2014). In addition, it is anticipated that elevated blood bicarbonate levels contribute to an improvement in insulin resistance. In a subset of a large population‐based cross‐sectional analysis, higher bicarbonate levels were associated with lower fasting insulin concentration, whereas lower bicarbonate levels were associated with insulin resistance (Farwell and Taylor 2008). Therefore, a reduction in DAL due to the alkaline nature of a bicarbonate‐rich mineral water is expected to improve insulin resistance. Moreover, cortisol levels, usually elevated in metabolic acidosis, are suggested to be involved in insulin resistance (Dolati et al. 2024). However, positive effects of bicarbonate‐rich mineral water consumption on cortisol levels have not been demonstrated until now. Murakami and colleagues (Murakami et al. 2015) reported no significant differences in cortisol levels following the consumption of bicarbonate‐rich mineral water and low mineralized mineral water in a small study conducted with healthy individuals. These null findings may result from the low intake of only 500 mL of mineral water per day. However, the results are confirmed by a small study conducted with eight healthy male individuals. In this study, glucocorticoid secretion, specifically free cortisol and free cortisone in 24‐h urine, was not altered following the consumption of bicarbonate‐rich mineral water in individuals with sodium chloride‐induced low‐grade metabolic acidosis (Buehlmeier et al. 2016) (study B). In contrast, markers of glucocorticoid secretion were reduced following the administration of potassium bicarbonate over 10 days (Buehlmeier et al. 2016) (study A).

In addition, other mechanisms may be affected by bicarbonate‐rich mineral water consumption. Elevated lactate levels, indicative of metabolic acidosis, were also suggested to promote insulin resistance by increasing hepatic gluconeogenesis (Consoli et al. 1990). However, a direct causal relationship between high dietary acid load and body lactate production has not been established (Williams, Heilbronn, et al. 2016), and there are currently no studies on mineral water demonstrating a reduction in lactate levels following the consumption of bicarbonate‐rich mineral water.

##### 4.1.1.2 Lipid Metabolism

The effect of mineral water with a high bicarbonate content on parameters related to lipid metabolism has been studied in several randomized controlled trials, predominantly showing favorable outcomes (Table 2). These studies examined the effects of mineral water consumption in acute trials with a single consumption of 500 mL and in long‐term trials with daily consumption of 500 to 2000 mL over a period of up to 8 weeks. A comprehensive evaluation was conducted to assess the parameters of lipid metabolism and the atherosclerotic risk profile in individuals with and without diagnosed lipid metabolism disorders (Figure S2). Among the identified studies, bicarbonate‐rich mineral water consumption resulted in beneficial effects on aspects of lipid metabolism, while there was no such effect in the control group (Capurso et al. 1999; Dragomiretska et al. 2023; Dragomiretska et al. 2020; Pérez‐Granados et al. 2010; Schoppen et al. 2004; Schoppen, Pérez‐Granados, Carbajal, et al. 2005; Toxqui et al. 2012). In addition, some studies reported beneficial effects for both bicarbonate‐rich mineral water and control water (Aslanabadi et al. 2014; Toxqui and Vaquero 2016b; Zair et al. 2013). However, there were also studies that did not observe beneficial effects (Corradini 2012; Dore et al. 2021; Murakami et al. 2015; Schorr et al. 1996).

**TABLE 2:** Effects of bicarbonate‐rich mineral water on lipid metabolism.

Author	Design target group	Intervention	Characteristics of mineral water/treatment Bicarbonate/day	Main results	
				Time effects^a^ (Bicarbonate group)	Group differences time × water interaction
**Long‐term studies**					
Schorr et al. (1996)	Cross‐over, randomized, double‐blind 21 healthy older (60–72 years) individuals	NaCl reduction (< 100 mmol/d) + 4 weeks (each) 3 different mineral water brands 1.5 L/d	Water A: HCO_3_, Na, Mg Water B: HCO_3_, Na, Cl Water C: low mineralized HCO_3_ Water A: 2975 mg/d Water B: 1318 mg/d Water C: 18 mg/d	**Blood** (not specified)	
				**LDL** (plasma): ↔ **TC** (plasma): ↔ **TG** (plasma): ↔ **HDL** (plasma): ↔	No sign. group differences
Capurso et al. (1999)	Cross‐over (first water A, later water B) 10 individuals with moderate hypercholesteremia	Diet stabilization (3 weeks) + 3 weeks (each) Mineral water (water A: “Montecatini Regina”) vs. tap water (water B) Standardized diet 750 mL/d (morning)	Water A: HCO_3_, Na, Mg Water B: not reported HCO_3_ Water A: 508 mg/d Water B: not reported	**13‐h overnight fasting blood**	
				**LDL** (serum): ↓ **TC** (serum): ↓ **TG** (serum): ↔ **HDL** (serum): ↔ **Cardiovascular risk index** (TC/HDL): ↓ **ApoB** (serum): ↓	**LDL** (serum): water A < water B **TC** (serum): water A < water B **TG** (serum) ↔ time **HDL** (serum) ↔ time **Cardiovascular risk index** (TC/HDL): water A < water B **ApoB** (serum): water A < water B
				**24‐h stool**	
				**Bile acids**: ↑	**Bile acids**: water A > water B
				**Gallbladder**	
				**Volume**: ↓ (−40% at 15 and 30 min)	**Volume**: water A < water B
Schoppen et al. (2004)	Cross‐over 18 postmenopausal women	2 months (each) 2 different mineral water brands 1 L/d	Water A: HCO_3_, Na, Cl Water B: low mineralized HCO_3_ Water A: 2094 mg/d Water B: 71 mg/d	**12‐h overnight fasting blood**	
				**LDL** (serum): ↓ **HDL** (serum): ↑ **TC** (serum): ↓ **TG** (serum): ↔ **Cardiovascular risk indices** (TC/HDL u. LDL/HDL): ↓ **sICAM‐1**: ↓ **sVCAM‐1**: ↓ **Apo A and B**: ↔ **ATP‐III 10‐year risk**: ↓	**LDL** (serum): water A < water B **HDL** (serum): water A > water B **TC** (serum): water A < water B **TG** (serum): n.s. group differences **Cardiovascular risk indices** (TC/HDL u. LDL/HDL): water A < water B **sICAM‐1**: water A < water B **sVCAM‐1**: water A < water B **Apo A and B**: n.s. group differences **ATP‐III 10‐year risk**: water A < water B
Pérez‐Granados et al. (2010)	Cross‐over (first water B, later water A), single‐blind 18 young (> 18–< 40 years) hypercholesterolemic individuals	8 weeks (each) 2 different mineral water brands 1 L/d	Water A: HCO_3_, Na, Cl Water B: low mineralized HCO_3_ Water A: 2120 mg/d Water B: 104 mg/d	**12‐h overnight fasting blood**	
				**LDL** (serum): ↓ **TC** (serum): ↓ **TG** (serum): ↔ **HDL** (serum): ↔ **Cardiovascular risk indices** (TC/HDL u. LDL/HDL): ↓ **Apo A**: ↔ **Apo B**: ↓ **sICAM‐1**: ↔ **sVCAM‐1**: ↔	**LDL** (serum): water A < water B **TC** (serum): water A < water B **TG** (serum): n.s. group differences **HDL** (serum): n.s. group differences **Cardiovascular risk indices** (TC/HDL u. LDL/HDL): water A < water B **Apo A**: n.s. group differences **Apo B**: water A < water B **sICAM‐1**: n.s. group differences **sVCAM‐1**: n.s. group differences
Corradini (2012)	Parallel‐group 40 postmenopausal women with functional dyspepsia and/or constipation	12 days Thermal water (33°C) (water A: “Aqua Santa di Chianciano Terme”) vs. tap water (water B) 500 mL/d (morning)	Water A: HCO_3_, Ca, Mg, SO_4_ Water B: low mineralized HCO_3_ Water A: 365 mg/d Water B: 0 mg/d	**Overnight fasting blood**	
				**LDL** (serum): ↔ **TC** (serum): ↔ **TG** (serum): ↔ **HDL** (serum): ↔ **Bile acids** (serum): ↑	No sign. group differences (lipids) **Bile acids** (serum): n.s. group differences
				**Gallbladder**	
				**Fasting volume**: ↓	**Fasting volume:** n.s. group differences
Zair et al. (2013)	Cross‐over, randomized, double‐blind 12 hypercholesterolemic men	8 weeks (each) 2 different mineral water brands (water A: “St. Yorre”; water B: “Ogeu”) 1.25 L/d	Water A: HCO_3_, Na, Cl Water B: low mineralized HCO_3_ Water A: 5210 mg/d Water B: 229 mg/d	**Fasting blood**	
				**LDL**: ↔ **TC** (plasma): ↔ **Cholesterol in VLDL:** ↔ (tendency ↓ *p* = 0.066)^b^ **HDL**: ↔ **TG** (plasma): ↓ **TG in VLDL**: ↓	No sign. group differences
Aslanabadi et al. (2014)	Parallel‐group, randomized 69 hyperlipidemic individuals	1 month 2 different mineral water brands 1 L/d	Water A: HCO_3_, Ca, Mg Water B: low mineralized mineral water HCO_3_ Water A: 1350 mg/d Water B: 29 mg/d	**Blood** (not specified)	
				**LDL**: ↓ **TC**: ↓ **TG** (plasma): ↔ **HDL** (plasma): ↔	No sign. group differences
Murakami et al. (2015)	Cross‐over 19 healthy individuals	2 × 1 week (each) Mineral water (water A) vs. tap water (water B) 500 mL/d (before meals)	Water A: HCO_3_, Na, Ca, Mg, SO_4_ Water B: low mineralized HCO_3_ Water A: 1243 mg/d Water B: 14 mg/d	**Fasting blood**	
				Not reported	No sign. group differences for **LDL** **TC** **TG** **HDL**
Toxqui and Vaquero (2016b)	Cross‐over, randomized, single‐blind 64 moderately hypercholesterolemic men and women	8 weeks (each) 2 different mineral water brands 1 L/d	Water A: HCO_3_, Na, Cl Water B: low mineralized HCO_3_ Water A: 2050 mg/d Water B: 75 mg/d	**12‐h overnight fasting blood**	
				**LDL**: ↓ **oxLDL**: ↔ **TC**: ↓ **TG**: ↔ **HDL** ↔ **Cardiovascular risk indices** (TC/HDL u. LDL/HDL): ↓ **LDL/Apo B**: ↓ **HDL/Apo A‐I**: ↔ **Apo A‐I**: ↔ **APO B‐100**: ↑	No sign. time × water interactions
Dragomiretska et al. (2020)	Parallel‐group, randomized 90 individuals with GERD	21–24 days (Basic therapy: proton pump inhibitors) Basic therapy vs. basic therapy +2 different mineral water brands (water A: “Polyana Kvasova”, water B: “Donat‐Mg”) 1% of body weight (three doses, before the meals; 450–690 mL/d)	Water A: HCO_3_, Na, B Water B: HCO_3_, Mg, Na, SO_4_ HCO_3_ both waters individualized	**Blood** (not specified)	
				Water A: n.s. time effects for functional state of the liver Water B: **TC**: ↓ **LDL**: ↓ **HDL**: ↔ **TG**: ↔ **Coefficient of atherogenicity**: ↔	Not reported
Dore et al. (2021)	Single‐arm 10 patients in hospital (internal medicine)	10 days Mineral water (“San Martino”) 1–2 L/d	Water: HCO_3_, Na, Cl, SO_4_ HCO_3_ (1,5 L) Water: 3432 mg/d	**Overnight fasting blood**	
				**TC:** ↔ **TG:** ↔	—
Dragomiretska et al. (2023)	Parallel‐group, randomized 71 men and women with chronic viral hepatitis C (concomitant nonalcoholic fatty liver disease), after Covid‐19 infection	2 months Diet + exercise (control) vs. diet + exercise + mineral water (“Shayanskaya”) 3 mL water/kg body weight, before and after meals (3 meals)	Water: HCO_3_, Na, Si HCO_3_ Individualized (1453 g/L)	**Blood** (not specified)	
				**LDL**: ↓ **TC**: ↓ **TG**: ↓ **HDL**: ↑ **Coefficient of atherogenicity**: ↓	**LDL**: water < control **TC**: n.s. group differences **TG**: n.s. group differences **HDL**: n.s. group differences **Coefficient of atherogenicity**: water < control
Jovicic et al. (2023)	Single‐arm 60 individuals with diagnosed type 2 diabetes mellitus	28 days Mineral water (“Sneznik‐1”) 1.5 to 3 L (depending on disease stage and general condition)	Water: HCO_3_ HCO_3_ Individualized	**Blood (not specified)**	
				**LDL**: ↔ **TC**: ↓ **TG**: ↔ **HDL**: ↔	—
**Acute studies**					
Marchi et al. (1992)	Cross‐over 10 individuals	— Mineral water (“Acqua di Uliveto”) vs. control solution (NaCl) 400 mL	Water: HCO_3_, Ca Control solution: NaCl	**Gallbladder**	
				**Volume**: sign. time effects	**Volume**: sign. group differences at 10 min, 20 min, 30 min, 40 min, 50 min, 60 min (water<NaCl)
Schoppen, Pérez‐Granados, Carbajal, et al. (2005)	Cross‐over, randomized 18 postmenopausal women	— 3 different mineral water brands Standardized meal (fat‐rich) 500 mL	Water A: HCO_3_, Na (higher than water B), Cl Water B: HCO_3_, Na, Cl Water C: low mineralized HCO_3_ Water A: 1047 mg Water B: 1007 mg Water C: 36 mg	**12‐h overnight fasting blood + postprandial**	
				**TC** (serum): n.s. time effect **Cholesterol in chylomicrons:** sign. time effect **TG** (serum): sign. time effect **TG in chylomicrons**: sign. time effect Area under the curve (AUC) — Peak concentration —	**TC** (serum): n.s. group differences **Cholesterol in chylomicrons:** n.s. group differences **TG** (serum): water B < water C **TG in chylomicrons**: n.s. group differences No sign. time × water interactions for all lipid parameters Area under the curve (AUC) **TC** (serum): n.s. group differences **Cholesterol in chylomicrons:** n.s. group differences **TG** (serum): water B < water C **TG in chylomicrons**: sign. group differences: waters A and B < water C (n.s.) Peak concentration **TC (serum)**: n.s. group differences **Cholesterol in chylomicrons:** n.s. group differences **TG (serum):** sign. group differences: waters A and B < water C (n.s.) **TG in chylomicrons**: n.s. group differences
Toxqui et al. (2012)	4 way cross‐over (with/without meal), randomized 21 young (> 18–< 40 years) moderately hypercholesteremic individuals	— 3 different mineral water brands Standardized meal (fat‐rich)/without meal 500 mL	Water A: HCO_3_, Na, Cl Water B: low mineralized HCO_3_ Water A: 1060 mg Water B: 52 mg	**12‐h overnight fasting blood** (+ postprandial)	
				**Without meal** **TG**: n.s. time effect **With meal** **TG**: sign. time effect **Cholecystokinin**: sign. time effect	**Without meal** **TG**: n.s. group differences **With meal** **TG**: water A < water B at 30 min, 60 min **Cholecystokinin**: water A < water B at 30 min
				**Gallbladder**	
				**Without meal** — **Volume**: sig. time effect **With meal** — **Volume**: sig. time effect	**Without meal** **Area under the curve (AUC)**: n.s. group differences **Ejection fraction**: n.s. group differences **Peak contraction**: n.s. group differences **Volume**: n.s. group differences **With meal** **Area under the curve (AUC)**: water A > water B **Ejection fraction**: water A < water **Peak contraction**: water A > water B **Volume**: water A > water B at 30 min, 60 min, 120 min
Zair et al. (2013)	Cross‐over, randomized, double‐blind 12 hypercholesteremic men	— Two separate postprandial cycles (before + after 8 weeks treatment) 2 different mineral water brands (water A: “St. Yorre”; water B: “Ogeu”) Standardized meal 500 mL	Water A: HCO_3_, Na, Cl Water B: low mineralized HCO_3_ Water A: 2084 mg Water B: 92 mg	**Fasting blood**	
				Area under the curve (AUC), between two time points **Cholesterol in VLDL**: ↔ **LDL**: ↔ **HDL**: ↔ **TG** (plasma): ↔ **TG in VLDL:** ↔	No sign. group differences

*Note:* Minerals: Ca, calcium; Cl, chloride; HCO_3_, bicarbonate; Mg, magnesium; Na, sodium; SO_4_, sulfate. Blood parameters: APO‐A, apolipoprotein A; APO‐B, apolipoprotein B; HDL, high‐density lipoprotein; LDL, low‐density lipoprotein; oxLDL, oxidized low‐density lipoprotein; sICAM, soluble intercellular adhesion molecule; sVCAM, soluble vascular adhesion molecule; TC, total cholesterol; TG, triglycerides; VLDL, very low‐density lipoprotein. ↑ = significant increase (*p* < 0.05); ↔ = no significant change (*p* > 0.05); ↓ = significant decrease (*p* < 0.05).

^a^ Between begin and end of each intervention period.

^b^ Categorization of the authors.

Studies that did not observe favorable effects on lipid metabolism tended to be of shorter duration, typically lasting between 10 days and 4 weeks (Corradini 2012; Dore et al. 2021; Jovicic et al. 2023; Murakami et al. 2015; Schorr et al. 1996). Furthermore, these studies utilized lower amounts of bicarbonate per day (Corradini 2012). Notably, Perez‐Granados and colleagues (Pérez‐Granados et al. 2010) reported no statistically significant changes after 4 weeks in moderately hypercholesterolemic individuals, whereas a significant effect was observed after 8 weeks of mineral water consumption. However, acute studies also reported positive effects in some aspects of lipid metabolism (Schoppen, Pérez‐Granados, Carbajal, et al. 2005; Toxqui et al. 2012). Given the presence of both beneficial and null findings in individuals with elevated lipid metabolism parameters at the baseline examinations, it can be concluded that these findings are not contingent on the subject's metabolic state at the beginning of the study (Figure S2).

Nearly all studies evaluating subchronic consumption of bicarbonate‐rich mineral water reported a significant reduction in **total cholesterol** (TC) and/or **low‐density lipoprotein cholesterol** (LDL cholesterol) in the bicarbonate groups. In detail, TC decreased by approximately 15% in individuals with diagnosed type 2 diabetes mellitus following a 28‐day mineral water consumption (Jovicic et al. 2023). Moreover, a total of five studies demonstrated the superiority of bicarbonate‐rich mineral water. One of these studies, which was conducted over the course of 8 weeks, involved the administration of 1000 mL of mineral water per day to postmenopausal women. The results of this study indicated a significant reduction in both LDL and total cholesterol, with a decrease of 14.8% and 6.8%, respectively, compared to the control phase (Schoppen et al. 2004). A comparable decrease of nearly 10.0% and 6.3%, respectively, was noted in individuals with hypercholesterolemia, as compared to the control phase (Pérez‐Granados et al. 2010). In accordance with these findings, LDL was reduced by 12.5%, and TC was reduced by 7.5%, in individuals with mild hypercholesterolemia who consumed 750 mL of mineral water in replacement of tap water (Capurso et al. 1999). Although no percentages were mentioned, a recent study on individuals with NAFLD also reported a reduction in LDL after the consumption of bicarbonate‐rich mineral water compared to a control group without mineral water consumption. The observed reduction in TC did not show any group differences (Dragomiretska et al. 2023). In addition, the same research group reported a significant reduction in TC and LDL in individuals with gastroesophageal reflux disease (GERD), receiving a basic therapy and a high mineralized mineral water rich in bicarbonate, magnesium, sodium, and sulfate. However, the second study arm receiving a sodium‐bicarbonate mineral water did not show significant changes in the lipid profile (Dragomiretska et al. 2020). It is not clear if the bicarbonate content of the two mineral waters differed, as no composition of the mineral waters was reported. Therefore, different amounts of bicarbonate consumption might have been the reason for the observed differences in lipid profile. Conversely, two studies did not support the hypothesis that bicarbonate‐rich mineral water is superior to other treatments, as both intervention groups exhibited significant reductions (Aslanabadi et al. 2014; Toxqui and Vaquero 2016b). The lack of evidence for the superiority in these two studies may be partly due to dietary factors. For instance, in the study by Toxqui and Vaquero (2016b), saturated fat intake was reduced in the control group but not in the bicarbonate group, potentially counterbalancing the effects of bicarbonate water. Similar dietary changes may have occurred in the study by Aslanabadi et al. (2014), where no information on diet was reported. However, acute consumption studies using 500 mL of mineral water alongside a high‐fat meal also failed to show positive results on LDL and total cholesterol levels (Schoppen, Pérez‐Granados, Carbajal, et al. 2005; Zair et al. 2013), possibly due to the single low consumption.

In addition, **triglyceride** (TG) levels were reduced by the consumption of bicarbonate‐rich mineral water. In two acute studies conducted with postmenopausal women and moderately hypercholesterolemic young adults, the consumption of 500 mL of bicarbonate‐rich mineral water alongside a high‐fat meal resulted in a significant reduction of postprandial TG levels (Schoppen, Pérez‐Granados, Carbajal, et al. 2005). Although no time × water interactions could be demonstrated, there was a significant difference in the area under the curve for serum triglycerides in one of these studies evaluating the effect of three different mineral waters on postprandial triglyceride levels. However, only one of the studied bicarbonate‐rich mineral waters led to a significantly lower area under the curve compared to the control water. In addition, chylomicron TG showed a significant water effect, although the differences between the waters were not significant. The authors attribute this to methodological steps in sample handling (Schoppen, Pérez‐Granados, Carbajal, et al. 2005). In the other acute study on triglyceride levels, a similar bicarbonate load via bicarbonate‐rich mineral water resulted in a significantly lower triglyceride level 30 min and 60 min after the consumption of a fat‐rich meal compared to a low mineralized mineral water (Toxqui et al. 2012). In addition, a significantly lower increase in cholecystokinin release, a hormone that regulates gallbladder emptying, was shown compared to the control water. This may be the reason for the lower measured gallbladder emptying and, in consequence, the lower TG levels, possibly due to a lower lipid absorption (Toxqui et al. 2012). However, only two studies have confirmed these findings on TG levels following subchronic consumption of bicarbonate‐rich mineral water (Dragomiretska et al. 2023; Zair et al. 2013). In the first study, plasma TG levels declined by 23% and levels of TG in very low‐density lipoprotein (VLDL) decreased by an even greater extent, amounting to 31%. However, no superiority of bicarbonate‐rich mineral water could be demonstrated, as TG levels in the intervention groups did not differ significantly within the respective study (Zair et al. 2013). In contrast, further studies evaluating chronic consumption of bicarbonate‐rich mineral water failed to demonstrate statistically significant changes in TG levels (Aslanabadi et al. 2014; Pérez‐Granados et al. 2010; Schoppen et al. 2004; Toxqui and Vaquero 2016b).

In terms of **high‐density lipoprotein cholesterol** (HDL cholesterol), only one singular study reported a favorable increase of 8.7% compared to the control group (Schoppen et al. 2004). Although another study showed a significant increase in the mineral water group, no superiority of bicarbonate‐rich mineral water could be demonstrated when the study groups were compared. In this study, changes in HDL possibly occurred as a consequence of adherence to the Mediterranean diet in both groups during the intervention period (Dragomiretska et al. 2023). This is in contrast to the stable HDL cholesterol levels observed in several other studies on this topic (Aslanabadi et al. 2014; Murakami et al. 2015; Pérez‐Granados et al. 2010; Schorr et al. 1996; Toxqui and Vaquero 2016b; Zair et al. 2013). Therefore, it seems rather unlikely that the consumption of bicarbonate‐rich mineral water has an effect on HDL levels.

However, additional beneficial effects on cardiovascular health have been observed in subchronic consumption studies. Toxqui and Vaquero (Toxqui and Vaquero 2016b) demonstrated improvements in **cardiovascular risk indices** (TC/HDL, LDL/HDL) in both the bicarbonate group and the control group of hypercholesterolemic individuals. Slightly differently, two studies evaluating the effects in postmenopausal and hypercholesterolemic individuals also reported reductions, with bicarbonate‐rich mineral water showing superiority over control water (Pérez‐Granados et al. 2010; Schoppen et al. 2004). While TC/HDL and LDL/HDL were reduced by 18.5% and 22.8% in the first study (Schoppen et al. 2004), both indices decreased by 7.5% and 11.5% in the second study (Pérez‐Granados et al. 2010) compared to the respective control group. Moreover, the coefficient of atherogenicity was reduced after the consumption of individualized mineral water intake (3 mL water/kg body weight), with a significantly lower value in the bicarbonate‐rich mineral water group compared to the control group over a consumption period of 2 months (Dragomiretska et al. 2023).

Findings regarding **inflammatory markers** that influence the risk of atherosclerosis are scarce and inconclusive. One study demonstrated an 8.4% reduction in soluble intercellular adhesion molecule‐1 (sICAM‐1) and a 14.8% reduction in soluble vascular cell adhesion molecule (sVCAM) levels compared to the control group in postmenopausal women (Schoppen et al. 2004). In contrast, these markers remained unaffected and stable in young hypercholesterolemic adults (Pérez‐Granados et al. 2010). This discrepancy may be attributable to factors independent of mineral water consumption, including age and subchronic inflammation.

###### Mechanisms

The proposed mechanisms underlying the reduction of LDL cholesterol/total cholesterol and postprandial lipemia/triglycerides differ in several aspects. A number of studies have postulated an intestinal osmotic effect of bicarbonate‐ and sodium‐rich mineral water, attributable to its elevated mineral content (Aslanabadi et al. 2014; Pérez‐Granados et al. 2010; Schoppen, Pérez‐Granados, Carbajal, et al. 2005). The osmotic effect is believed to reduce cholesterol absorption, leading to lower cholesterol levels. In addition, the alkalinizing effect of the bicarbonate‐rich mineral water induced intestinal pH changes (Lu et al. 2022; Pérez‐Granados et al. 2010; Toxqui and Vaquero 2016b; Wasserfurth et al. 2019; Wynn, Krieg, et al. 2009). However, gastric alterations are also possible. As a result of an increase in intestinal pH, the absorption of cholesterol may be reduced (Schoppen, Pérez‐Granados, Carbajal, et al. 2005; Toxqui et al. 2012). Another aspect discussed in the literature is the effect on the human bile acid pool. Increased fecal bile acid excretion, possibly due to a reduced bile acid reabsorption, may contribute to a higher conversion rate of cholesterol into bile acids via 7α‐hydroxylase, resulting in lower cholesterol levels (Capurso et al. 1999; Schoppen, Pérez‐Granados, Carbajal, et al. 2005). This hypothesis is based on an older study demonstrating a 98% increase in fecal bile acid excretion following a 3‐week consumption of sodium‐rich mineral water with an average bicarbonate content (Capurso et al. 1999). However, it is worth noting that the bicarbonate content of the mineral water studied was relatively low (677 mg/L). Therefore, the observed effect on bile acid excretion may have been primarily due to the high sodium content rather than the bicarbonate content or the alkalinizing effect of the mineral water. Nevertheless, this increased loss of bile acids has been attributed to a rapid contraction of the gallbladder, leading to a faster transit time of the intestinal contents due to the large influx of bile into the duodenum (Capurso et al. 1999). This aligns with findings demonstrating a reduced gallbladder volume following the consumption of bicarbonate‐rich mineral water in an acute consumption study (Marchi et al. 1992).

In contrast, there is also one study showing a higher gallbladder volume (Toxqui et al. 2012) following the consumption of bicarbonate‐ and sodium‐rich mineral water compared to individuals drinking a low mineralized control water. In this study, it was hypothesized that the large gallbladder volume would result in reduced lipid absorption due to decreased bile acid secretion as a result of a lower gallbladder ejection fraction. Furthermore, it was observed that cholecystokinin (CCK), a hormone regulating gallbladder contraction, decreased following the consumption of the respective mineral water. Consequently, this reduction in gallbladder contraction led to a decrease in bile acid levels in the intestinal tract (Toxqui et al. 2012). The authors hypothesized that the alkaline nature of the bicarbonate mineral water may inhibit lipase activity, thereby reducing triglyceride hydrolysis and suppressing CCK release.

##### 4.1.1.3 Blood Pressure

The effect of bicarbonate‐ and sodium‐rich mineral water on blood pressure has been evaluated in acute trials with a single consumption of 500 mL and in studies evaluating the long‐term consumption of 500 mL to 3000 mL per day over a consumption period of up to 8 weeks (Table 3). In these studies, no adverse effects of bicarbonate‐ and sodium‐rich mineral water consumption on blood pressure have been reported (Schoppen et al. 2004; Schorr et al. 1996; Zair et al. 2013; Toxqui and Vaquero 2016b; Dore et al. 2021; Mansouri et al. 2023). However, even a favorable change in blood pressure has been demonstrated (Luft et al. 1990; Pérez‐Granados et al. 2010).

**TABLE 3:** Effects of bicarbonate‐rich mineral water on blood pressure.

Author	Design target group	Intervention	Characteristics of mineral water/treatment Bicarbonate/day	Main results	
				Time effects^a^ (Bicarbonate group)	Group differences time × water interaction
**Long‐term studies**					
Luft et al. (1990)	Cross‐over, randomized, single‐blind 10 individuals (hypertensive + normotensive)	4 days Run‐In +7 days (each) Mineral water (“Staatl. Fachingen”) vs. control solution (NaCl) Standardized diet (low sodium, low calcium) 3 L/d	Water: HCO_3_, Na, Mg Control solution: Na, Cl, Mg HCO_3_ Water A: 6046 mg/d Water B: 0 mg/d	**Blood pressure measurements**	
				**SBP:** ↓ (hypertensives) ↔ (normotensives) **DBP**: ↔ (normo/hypertensives)	Not reported
				**Urine** (not specified)	
				**Na**: ↑	**Na**: n.s. group differences (treatment groups, normo/hypertensives, black/white)
				**Fasting blood**	
				**Renin** (plasma): ↓ (hypertensives) ↔ (normotensives) **Aldosterone** (plasma): ↔	**Renin** (plasma): n.s. group differences (hypertensives) **Aldosterone** (plasma): not reported
Schorr et al. (1996)	Cross‐over, randomized, double‐blind 21 healthy older (60–72 years) individuals	NaCl reduction (< 100 mmol/d) + 4 weeks (each) 3 different mineral water brands 1.5 L/d	Water A: HCO_3_, Na, Mg Water B: HCO_3_, Na, Cl Water C: low mineralized HCO_3_ Water A: 2975 mg/d Water B: 1318 mg/d Water C: 18 mg/d	**Blood pressure measurements**	
				**24‐h BP**: ↔ **MAP**: ↓ (water A and water C) ↔ (water B)	**24‐h BP**: n.s. group differences **MAP** water B > water C
				**24‐h urine**	
				**Na:** ↑ (water B)	Week 4 **Na**: water A and B > than water C; water A < water B
				**Blood** (not specified)	
				**Aldosterone** (plasma): ↔ **Renin** (plasma): ↔ **ANP** (plasma): ↔	Week 4 No sign. group differences
Schoppen et al. (2004)	Cross‐over 18 postmenopausal women	2 months (each) 2 different mineral water brands 1 L/d	Water A: HCO_3_, Na, Cl Water B: low mineralized HCO_3_ Water A: 2094 mg/d Water B: 71 mg/d	**Blood pressure measurements**	
				**SBP**: ↔ **DBP**: ↔	No sign. group differences
Pérez‐Granados et al. (2010)	Cross‐over (first water B, later water A), single‐blind 18 young (> 18–< 40 years) hypercholesteremic individuals	8 weeks (each) 2 different mineral water brands 1 L/d	Water A: HCO_3_, Na, Cl Water B: low mineralized HCO_3_ Water A: 2120 mg/d Water B: 104 mg/d	**Blood pressure measurements**	
				**SBP**: ↓ **DBP**: ↔	**SBP**: water A < water B **DBP**: n.s. group differences
				**24‐h urine**	
				**Na**: ↑	Not reported
Zair et al. (2013)	Cross‐over, randomized, double‐blind 12 hypercholesteremic men	8 weeks (each) 2 different mineral water brands (water A: “St. Yorre”; water B: “Ogeu”) 1.25 L/d	Water A: HCO_3_, Na, Cl Water B: low mineralized HCO_3_ Water A: 5210 mg/d Water B: 229 mg/d	**Blood pressure measurements**	
				**SBP**: ↔ **DBP**: ↔	No sign. group differences
Murakami et al. (2015)	Cross‐over 19 healthy individuals	2 × 1 weeks (each) Mineral water (water A) vs. tap water (water B) 500 mL/d (before meals)	Water A: HCO_3_, Na, Ca, Mg, SO_4_ Water B: low mineralized HCO_3_ Water A: 1243 mg/d Water B: 14 mg/d	**Fasting blood**	
				Not reported	**Cortisol**: n.s. group differences
Buehlmeier et al. (2016)	Cross‐over, randomized 8 young, healthy men	14 days (each) High NaCl diet + mineral water (water A) vs. low NaCl diet + nonalkalizing drinking water (water B) Amount not reported	Water A: rot reported (HCO_3_‐rich) Water B: nonalkalizing HCO_3_ Water A: not reported Water B: not reported	**24‐h urine**	
				Markers for cortisol secretion **THE + THF + aTHF** (sum): ↔ Excretion of potentially bioactive free glucocorticoids **UFF + UFE** (sum): ↔ **UFF:** ↑ **UFE:** ↔ **UFE**/**UFF:** ↓	Markers for cortisol secretion **THE + THF + aTHF** (sum): n.s. group differences Excretion of potentially bioactive free glucocorticoids **UFF + UFE** (sum): n.s. group differences **UFF**: water A > water B **UFE**: **UFE**: n. s. group differences **UFE**/**UFF**: water A < water B
Toxqui and Vaquero (2016b)	Cross‐over, randomized, single‐blind 64 moderately hypercholesteremic men and women	8 weeks (each) 2 different mineral water brands 1 L/d	Water A: HCO_3_, Na, Cl Water B: low mineralized HCO_3_ Water A: 2050 mg/d Water B: 75 mg/d	**Blood pressure measurements**	
				**SBP**: ↔ **DBP**: ↔	No sign. time × water interactions
				**12‐h overnight fasting period**	
				**Aldosterone** (serum): ↔	No sign. time × water interaction
				**First morning urine**	
				**Na/creatinine**: ↔	No sign. time × water interaction
Dore et al. (2021)	Single‐arm 10 patients in hospital (internal medicine)	10 days Mineral water (“San Martino”) 1–2 L/d	Water: HCO_3_, Na, Cl, SO_4_ HCO_3_ (1,5 L) Water: 3432 mg/d	**Blood pressure measurements**	
				**SBP**: ↔ **DBP**: ↔	—
Mansouri et al. (2023)	Parallel‐group, randomized 94 healthy individuals	4 weeks 2 different mineral water brands 1.5–2 L/d	Water A: HCO_3_, Na, Cl Water B: low mineralized HCO_3_ (1,5 L) Water A: 6552 mg/d Water B: 342 mg/d	**Blood pressure measurements**	
				**SBP**: ↔ **DBP**: ↔ **MAP**: ↔	**SBP**: n.s. time × water interaction (whole group) n.s. treatment × group interaction (normo‐vs. hypertensives) **DBP**: n.s. time × water interaction (whole group) n.s. treatment × group interaction (normo‐vs. hypertensives) **MAP**: n.s. time × water interaction (whole group) n.s. treatment × group interaction (normo‐vs. hypertensives)
				**12‐h overnight fasting blood**	
				**Aldosterone:** ↓	**Aldosterone**: water A < water B
				**24‐h urine**	
				**Na**: ↑	**Na**: water A > water B
**Acute studies**					
Schoppen et al. (2008)	Cross‐over, randomized 18 postmenopausal women	— 3 different mineral water brands 500 mL	Water A: HCO_3_, Na (higher than water B), Cl Water B: HCO_3_, Na, Cl Water C: low mineralized HCO_3_ Water A: 1047 mg Water B: 1007 mg Water C: 36 mg	**12‐h overnight fasting blood + postprandial**	
				**Aldosterone:** sign. time effect	**Aldosterone:** sign. group differences at 120 min (water A and B < water C); n.s. time × water interaction
				**Postprandial urine**	
				—	**Na:** water A > water C; *water A = water B*
Toxqui and Vaquero (2016a)	4 way cross‐over (with/without meal), randomized 21 young (> 18–< 40 years) moderately hypercholesteremic individuals	— 2 different mineral water brands Standardized meal (fat‐rich/without meal) 500 mL	Water A: HCO_3_, Na, Cl Water B: low mineralized HCO_3_ Water A: 1060 mg Water B: 52 mg	**12‐h overnight fasting blood + postprandial**	
				**Without meal/with meal** **Aldosterone:** sign. time effect	**Without meal/with meal** **Aldosterone:** sign group differences at 30 min, 60 min and 120 min (water A < water B); higher reductions in women compared to men Differences between waters higher without meal

*Note:* Minerals: Ca, calcium; Cl, chloride; HCO_3_, bicarbonate; Mg, magnesium; Na, sodium; SO_4_, sulfate. Urinary parameters: aTHF, 5α tetrahydrocortisol; Na, sodium; THE, tetrahydrocortisone; THF, tetrahydrocortisol; UFE, free cortisone; UFF, Urinary free cortisol. Blood parameters: ANP, atrial natriuretic peptide. ↑ = significant increase (*p* < 0.05); ↔ = no significant change (*p* > 0.05); ↓ = significant decrease (*p* < 0.05).

^a^ Between begin and end of each intervention period.

The mineralogical composition of mineral water is influenced by the specific geological conditions prevalent in the region of origin. In certain instances, mineral water, characterized by a high bicarbonate content, exhibits a comparatively high sodium concentration. A review of 150 mineral waters revealed a negative correlation between the sodium content and the potential renal acid load (PRAL) in alkalizing mineral waters (Wynn, Raetz, and Burckhardt 2009). Given that mineral waters with a beneficial effect on acid–base balance are characterized by their alkalizing properties, mineral water with a low PRAL value often contains high sodium amounts. Consequently, the consumption of bicarbonate‐rich mineral water frequently coincides with elevated sodium levels and may affect blood pressure in salt‐sensitive individuals.

Nevertheless, there is a substantial body of evidence that resting blood pressure is stable following the consumption of bicarbonate‐ and sodium‐rich mineral water, despite the additional daily sodium intake from the mineral water ranging from 2562 to 3416 mg/d. In these studies, neither systolic blood pressure (SBP) nor diastolic blood pressure (DBP) was significantly affected by mineral water consumption (Dore et al. 2021; Mansouri et al. 2023; Schoppen et al. 2004; Toxqui and Vaquero 2016b; Zair et al. 2013). **Mean arterial pressure** (MAP) was reported in only two studies, yielding inconsistent results at first glance. In an earlier study, resting MAP decreased from baseline to the end of the study after the consumption of bicarbonate‐ and sodium‐rich mineral water as well as after low mineralized mineral water. The consumption of bicarbonate‐ and sodium chloride‐rich mineral water did not result in such changes. However, it should be taken into account that there was a salt reduction right after the beginning of the study, which induced a blood pressure reduction in all groups. Until the end of the study, blood pressure rose again in the group consuming bicarbonate and chloride‐rich mineral water, while it remained at a reduced level in the other groups (Schorr et al. 1996). Thus, the reported decline in MAP was not attributed to the mineral water consumption itself but to the salt restriction. In line with this, in a recent study, MAP remained stable following the consumption of bicarbonate‐ and sodium‐rich mineral water over a 4‐week period (Mansouri et al. 2023).

It is noteworthy that most studies did not perform subgroup analyses for hypertensive versus normotensive, male versus female, young versus old, or black versus white individuals. In addition, only one study examined the number of cases that experienced a change in blood pressure, which could reveal possible adverse effects in a subgroup (Mansouri et al. 2023). In this study, the majority of participants showed a stable or decreased blood pressure following the consumption of both bicarbonate‐ and sodium‐rich mineral water and low mineralized mineral water. However, a minority experienced an increase in blood pressure in both groups, with no statistically significant difference in the number of cases between the two mineral waters tested. Moreover, no differences in SBP, DBP, and MAP were observed between males and females or between younger (< 50 years) and older (≥ 50 years) individuals (Mansouri et al. 2023). In contrast, two older studies documented a decrease in SBP following the consumption of bicarbonate‐ and sodium‐rich mineral water. One of these studies reported a reduction in SBP in the entire study group (Pérez‐Granados et al. 2010), while the other showed a significant reduction only in hypertensive individuals (Luft et al. 1990).

**FIGURE 3:**
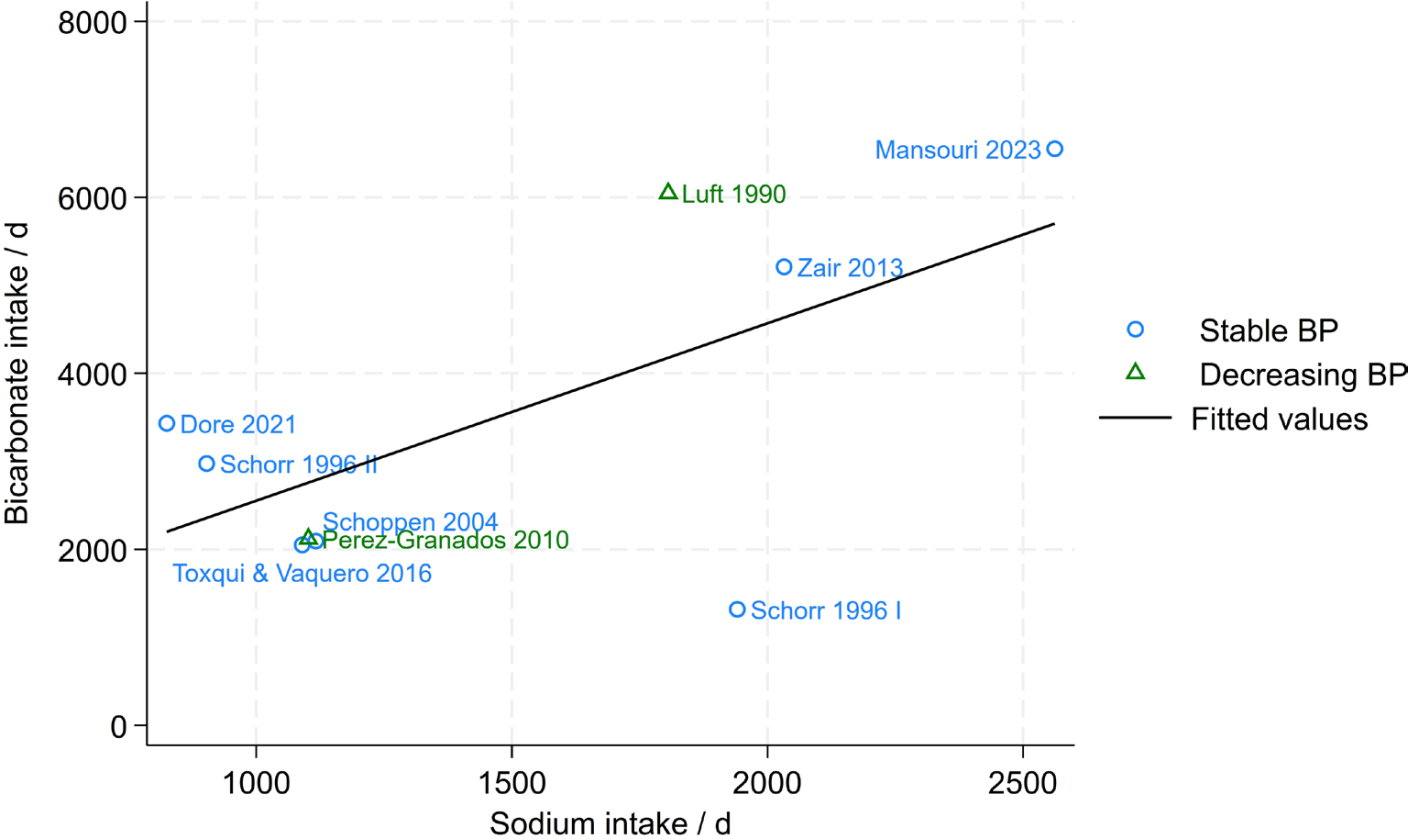
Associations between sodium and bicarbonate intake in intervention studies with bicarbonate‐rich mineral water.

Taken together, the data indicate that elevated sodium levels associated with the consumption of sodium‐bicarbonate‐rich mineral water do not result in a detrimental impact on blood pressure, regardless of the amount of sodium consumed (Figure 3).

###### Mechanisms

One of the underlying mechanisms for blood pressure changes as a reaction to high acidifying diets was already mentioned above: the involvement of cortisol. However, the data on the effect of bicarbonate‐rich mineral water consumption on cortisol levels is scarce, showing no beneficial effects (Buehlmeier et al. 2016; Murakami et al. 2015) (study B). This may result from the low daily consumption in these studies.

A second potential mechanism of blood pressure effects related to bicarbonate‐ and sodium‐rich mineral water involves the renin‐angiotensin‐aldosterone pathway. Acute studies have demonstrated a significant decrease in serum aldosterone (Schoppen et al. 2008; Toxqui and Vaquero 2016a), indicating a potential involvement of this pathway in maintaining stable blood pressure. This suggests efficient excretion of excess sodium in these studies. However, results from long‐term studies are inconclusive. Several studies conducted over periods of up to 8 weeks did not show a significant reduction in serum aldosterone (Luft et al. 1990; Schorr et al. 1996; Toxqui and Vaquero 2016b), although one of them demonstrated a reduction in the first study week, which disappeared until the end of the study (Schorr et al. 1996). In contrast, a recent 4‐week intervention study using bicarbonate‐ and sodium‐rich mineral water demonstrated a significant decrease in serum aldosterone compared to baseline, which was more pronounced in the bicarbonate group. This reduction possibly resulted from the high daily sodium intake inert in the studied mineral water (Mansouri et al. 2023).

A recent review has highlighted the importance of dietary chloride in blood pressure regulation, thus questioning the conventional emphasis on sodium content alone (Sadki et al. 2025). It is well‐established that sodium chloride has a detrimental effect on blood pressure in individuals who are salt‐sensitive (Bailey and Dhaun 2024). However, as described above, studies have shown that sodium bicarbonate, when consumed in mineral water, does not result in any adverse effects on blood pressure (Dore et al. 2021; Mansouri et al. 2023; Schoppen et al. 2004; Toxqui and Vaquero 2016b; Zair et al. 2013). This discrepancy underscores the necessity of considering the accompanying anion in sodium compounds. It is important to note that emerging evidence indicates a more specific and significant role for the chloride component of NaCl than sodium itself in salt‐sensitive blood pressure (Sadki et al. 2025). In this context, Luft et al. (1990) demonstrated that beverages differing in chloride concentration exerted varying effects on blood pressure among hypertensive patients, despite containing equivalent sodium levels. These findings underscore the pivotal role of the sodium anion in regulating blood pressure responses. Furthermore, McCallum's (McCallum et al. 2015) hypothesis suggests that the impact of chloride content on the renin‐angiotensin‐aldosterone system (RAAS) may exceed that of sodium, thereby underscoring the significance of chloride in dietary considerations for hypertension management.

### 4.2 Effects of Bicarbonate‐Rich Mineral Water on the Gastrointestinal Tract

Functional gastrointestinal disorders (FGIDs) are the most common diagnosis in gastroenterology (Drossman 2016). Results from a recent large‐scale multinational study of FGIDs suggest a prevalence between 20.7% and 40.3% for at least one FGID, depending on the type and manner of data collection (Sperber et al. 2021). Underlying processes combine motility disturbances, visceral hypersensitivities, altered mucosal and immune function, altered gut microbiota, and altered central nervous system processing. According to the Rome IV classification, FGIDs are divided into six groups based on the occurrence in different anatomical regions: (A) esophageal disorders, (B) gastroduodenal disorders, (C) bowel disorders, (D) centrally mediated disorders of gastrointestinal pain, (E) gallbladder and sphincter of Oddi disorders, (F) anorectal disorders.

#### 4.2.1 Diseases of the Upper Gastrointestinal Tract

Functional dyspepsia and functional heartburn are among the leading FGIDs of the upper gastrointestinal tract, along with functional dysphagia (Sperber et al. 2021). Symptoms include nausea, vomiting, acute abdominal pain or burning, bloating, abdominal distension, postprandial fullness, and early satiation (Drossman 2016; Pohl et al. 2016). Conventional treatment is often focused on the use of drugs inhibiting gastric acid secretion (proton pump inhibitors, H_2_ blockers) or neutralizing gastric acid (antacids). However, the relief in pain is often incomplete (Bertoni et al. 2002; Gasbarrini 2006; Labenz et al. 2023; Mönkemüller and Malfertheiner 2006), and longer drug use is accompanied by side effects (Gasbarrini 2006). As an alternative to drug treatment, bicarbonate‐rich mineral water has been used for several decades to treat upper gastrointestinal complaints, mainly dyspepsia and heartburn.

##### 4.2.1.1 Functional Dyspepsia

**FIGURE 4:**
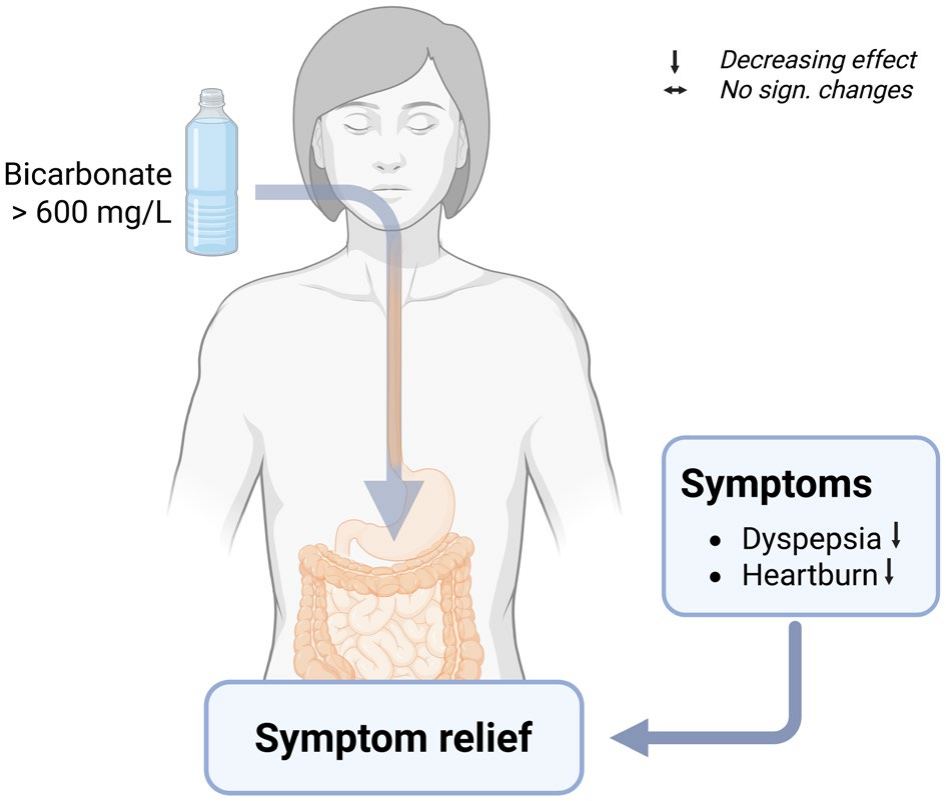
Effects of bicarbonate‐rich mineral water on diseases of the upper gastrointestinal tract. Created with BioRender.

The effect of bicarbonate‐rich mineral water on symptoms of functional dyspepsia has been evaluated in a few mineral water studies, showing positive results in symptom relief (Figure 4, Table 4). Evidence for a beneficial effect is provided by single‐arm and multiple‐arm trials. Mineral water consumption ranged from 500 mL/day for a consumption period of 8 days to 2000 mL/day for a period of 3 weeks. Some of the studies tested the effects only in individuals suffering from dyspeptic symptoms, while others tested the effects both on healthy individuals and individuals with irritable bowel syndrome (IBS) and/or dyspeptic complaints.

**TABLE 4:** Effects of bicarbonate‐rich mineral water on diseases of the upper‐gastrointestinal tract.

Author	Design target group	Intervention	Characteristics of mineral water Bicarbonate/day	Main results from bicarbonate group (time effects)^a^ and Group differences (if reported)
**Long‐term studies on dyspepsia**				
Bertoni et al. (2002)	Single‐arm 18 individuals with dyspepsia	30 days Mineral water 1.5 L/d	Water: HCO_3_, Ca HCO_3_ Water: 1025 mg/d	**Dyspeptic symptoms** ↓ **Frequency and severity of symptoms**: ↓
Gasbarrini (2006)	Parallel‐group 3872 individuals with dyspepsia Subgroup analyses for gastric output, GIT‐symptoms (60 dyspepsia individuals, 60 healthy controls)	3 × 21 days (21 days intervention +2 × 21 days follow‐Up) Different thermal water brands 2 L/d	Water‐group A: HCO_3_ Water‐group B: NaCl Water‐group C: SO_4_ HCO_3_ Not reported	No differentiation between waters Number of hospitalizations ↓ Days of absence from work ↓ Clinical recrudescence ↓ Subgroup analysis – **Prevalence of symptoms**: ↓, ↔ – **Gastric output**: ↓
Rocca et al. (2007)	Single‐arm (sequential trial) 29 individuals with functional dyspepsia	12 days Thermal water (33°C) (“Chianciano thermal water”) 200–400 mL/d (before breakfast)	Water: HCO_3_, Ca, Mg, SO_4_ HCO_3_ (300 mL) Water: 219 mg/d	**Global symptom score**: ↓ **Functional dyspepsia symptoms**: ↓ **Intensity of symptoms**: ↓ to a level not interfering with everyday activity **Associated symptoms** (heartburn and abdominal syndrome): ↔
Dragomiretska et al. (2020)	Parallel‐group, randomized 90 individuals with GERD	21–24 days Basic therapy: proton pump inhibitors Basic therapy vs. basic therapy +2 different mineral water brands (water A: “Polyana Kvasova,” water B: “Donat‐Mg”) 1% of body weight (three doses, before the meals; 450–690 mL/d)	Water A: HCO_3_, Na, B Water B: HCO_3_, Mg, Na, SO_4_ HCO_3_ both waters individualized	**water A** **Clinical symptoms:** ↓ **Acid‐forming function of the stomach**: normalization **water B** **Clinical symptoms**: ↓ **Acid‐forming function of the stomach**: normalization No group differences reported
Dore et al. (2021)	Single‐arm 10 patients in hospital (internal medicine)	10 days mineral water (“San Martino”) 1–2 L/d	Water: HCO_3_, Na, Cl, SO_4_ HCO_3_ (1,5 l) Water: 3432 mg/d	**Dyspepsia symptoms**:↓
**Long‐term studies on heartburn**				
Beer (2016)	Single‐arm 50 individuals with heartburn	1 week run‐in +6 weeks Mineral water 1.5 L/d (3 × 300 mL at meals, remaining volume in between)	Water: HCO_3_, Ca, Mg HCO_3_ Water: 2663 mg/d	**Occurrence of heartburn**: – Frequency of symptoms ↓ – Duration of symptoms ↓ **Reflux Disease Questionnaire**: symptom scores ↓ **Quality of life (QOLRAD)**: ↑ **Quality of life (GIQLI)**: ↑ **Health‐related quality of life (SF‐12)**: ↑
Pohl et al. (2016)	Single‐arm 56 individuals with heartburn	6 weeks Mineral water (“Staatl. Fachingen STILL”) 1.5 L/d (15–30 min before meals)	Water: HCO_3_, Mg, Na HCO_3_ Water: 2769 mg/d	**Occurrence of heartburn**: – Frequency of episodes↓ – Duration of episodes ↓ **Reflux Disease Questionnaire:** symptom scores *↓* **Quality of life (QOLRAD)**: ↑ **Quality of life (GIQLI)**: ↑ **Health‐related quality of life (SF‐12):** ↑
Dragomiretska et al. (2020)	Parallel‐group, randomized 90 individuals with GERD	21–24 days Basic therapy for all individuals: (proton pump inhibitors) Basic therapy vs. basic therapy +2 different mineral water brands (water A: “Polyana Kvasova,” water B: “Donat‐Mg”) 1% of body weight (three doses, before the meals; 450–690 mL/d)	Water A: HCO_3_, Na, B Water B: HCO_3_, Mg, Na, SO_4_ HCO_3_ Both waters individualized	**Water A** Heartburn: ↓ **Water B** Heartburn: almost elimination No group differences reported
Labenz et al. (2023)	Parallel‐group, randomized, double‐blind 148 individuals with heartburn Phase‐III‐Study	42 days 2 different mineral water brands (water A: “Staatl. Fachingen STILL”, water B: control) 1.5 L/d	Water A: HCO_3_, Mg Water B: low mineralized HCO_3_ Water A: 2769 mg/d Water B: approx. 45 mg/d	**Responder rate**: 84.7% **Reflux Disease Questionnaire** (RDQ): – Heartburn score ↓ (verum > control) – Regurgitation score ↓ (verum = control) – Dyspepsia score ↓ (verum = control) **Quality of life** (QOLRAD): ↑ – Problems with eating and drinking ↑ (verum > control) – Emotional distress score ↑ (verum > control) – Vitality ↑ (verum > control) – Sleeping disturbance ↑ (verum = control) – Physical/social functioning ↑ (verum = control) **Rescue medication**: ↓ (verum > control) Placebo‐corrected treatment effect: > 20%

*Note:* Minerals: Ca, calcium; Cl, chloride; HCO_3_, bicarbonate; Mg, magnesium; Na, sodium; SO_4_, sulfate. Questionnaires: GIQLI, Gastrointestinal Quality of Life index; QOLRAD, Quality Of Life in Reflux and Dyspepsia; RDQ, Reflux Disease Questionnaire; SF‐12, Short‐form 12. ↑ = significant increase (*p* < 0.05); ↔ = no significant change (*p* > 0.05); ↓ = significant decrease (*p* < 0.05).

^a^ Between begin and end of each intervention period.

Beneficial effects of mineral water were demonstrated in a large cohort of patients (*n* = 3872) suffering from dyspeptic symptoms. In this intervention study, individuals consumed 2000 mL of one of three different mineral waters (bicarbonate‐rich, salt‐rich, sulfate‐rich) per day in up to three cycles. Treatment cycles were separated by one year and lasted 3 weeks each. The consumption of mineral water led to a significant reduction in the prevalence of dyspeptic symptoms (epigastric pyrosis, postprandial fullness, bloating), evaluated in a subgroup of 60 participants. In addition, epigastric pain, nausea, and vomiting were reduced in several individuals, but this reduction did not reach statistical significance for the whole group (Gasbarrini 2006). However, the data on the effects were evaluated in a combined manner (all water groups together), which does not allow conclusions to be drawn about different mineral waters. Moreover, the authors did not specify the mineral content of the mineral waters used by stating the average amount of minerals. It is therefore impossible to estimate which ingredient or combination of ingredients might have caused the effects. However, similar results were obtained in a single‐arm sequential trial using 200 to 400 mL of thermal water with a medium bicarbonate content of 730 mg/L. In this study, epigastric pain and epigastric burning were reduced. In addition, the consumption of bicarbonate‐rich mineral water showed beneficial effects regarding postprandial fullness, early satiety, gastric distension, nausea, and vomiting (Rocca et al. 2007). In this context, it is noteworthy that the thermal water contained high levels of sulfate and calcium. Thus, the beneficial effects could have resulted from the high ingestion of these minerals. In a further study, the consumption of 1500 mL bicarbonate‐ and calcium‐rich mineral water per day led to a significant reduction in frequency and severity of epigastric pain, retrosternal pyrosis, postprandial fullness and feeling of gastric distension after a consumption period of 30 days. Moreover, epigastric burning was positively affected. However, only the severity of epigastric burning was improved, while frequency was not affected (Bertoni et al. 2002). In line with these findings, the consumption of 1000 mL to 2000 mL per day of a different bicarbonate‐rich mineral water showed symptom relief in 80% of the enrolled dyspeptic individuals, leading to a normalization of digestive function (Dore et al. 2021). Similar results were reported in a study evaluating the influence of two different bicarbonate‐rich mineral waters (1: rich in bicarbonate and boron, 2: rich in bicarbonate, sulfate, and magnesium) compared to a control group without mineral water consumption in individuals with GERD (Dragomiretska et al. 2020). Both intervention groups and the control group underwent basic treatment with proton pump inhibitors. After 3 weeks of mineral water consumption, a reduction in clinical symptoms occurred. In detail, epigastric pain, heartburn, and nausea were reduced. However, the mineral water high in bicarbonate, sulfate, and magnesium exhibited a more significant effect than the mineral water with a high bicarbonate, sodium, and boron content, completely eliminating epigastric pain. Additionally, consumption of this mineral water led to notable reductions in belching, pain in the right hypochondrium, and bitter taste. Moreover, constipation decreased to zero in this group, potentially due to the elevated magnesium and sulfate levels of that specific mineral water. In all three groups, the gastric pH values were reduced due to the basic treatment with proton inhibitors. While most individuals in the control group achieved hypoacidity after a stimulation test, almost all individuals in the mineral water groups returned to normal acidity (Dragomiretska et al. 2020).

###### Mechanisms

Individuals suffering from dyspepsia show a delayed gastric emptying rate possibly due to reduced gastric motility (Gasbarrini 2006; Madisch et al. 2018). Moreover, increased sensitivity to gastric acid, with or without inflammation of the mucosa may cause epigastric pain (Bertoni et al. 2002).

In mineral water studies, it was demonstrated that the consumption of bicarbonate‐rich mineral water improves parameters of gastric emptying (Bertoni et al. 2002; Gasbarrini 2006). The bicarbonate content in these mineral waters has been shown to enhance the gastric pH (Grassi et al. 1993). This, in turn, is believed to affect the secretion of digestive hormones such as gastrin (Bertoni et al. 2002; Gasbarrini 2006), which has motor activation potentials (Mearadji et al. 1999). Indeed, the use of a selective antagonist of the gastrin receptor inhibited the activation of gastric function observed after the consumption of bicarbonate‐rich mineral water in a preclinical study on rats. The authors concluded that bicarbonate‐rich mineral water may increase gastrin levels, which could explain the observed digestive effects (Bertoni et al. 2002). However, some of the studied mineral waters on dyspeptic symptoms were characterized by a high bicarbonate and a high calcium content. Calcium is believed to partially mitigate heartburn by enhancing peristalsis and acid clearance (Rodriguez‐Stanley et al. 2004). Therefore, it's important to acknowledge the potential additional effect of calcium ions (Pohl et al. 2016).

##### 4.2.1.2 Heartburn

In the past decades, the effect of bicarbonate‐rich mineral water on heartburn has been evaluated in a few mineral water studies, showing favorable results regarding the improvement of symptoms (Table 4). Evidence supporting a beneficial effect is obtained from single‐arm studies (Beer 2016; Pohl et al. 2016) and studies in a parallel‐group design (Dragomiretska et al. 2020; Labenz et al. 2023). In these studies, mineral water consumption ranged from 450 mL over a period of approximately 3 weeks (Dragomiretska et al. 2020) up to 1500 mL/day for a consumption period of 6 weeks (Beer 2016; Labenz et al. 2023; Pohl et al. 2016). All these studies were conducted with participants experiencing recurrent episodes of heartburn lasting at least three or 6 months before the study entry.

As mentioned above in the dyspepsia section, a Ukrainian study demonstrated the beneficial effects of two bicarbonate‐rich mineral waters on individuals diagnosed with GERD. In this intervention study, both mineral waters reduced heartburn; however, one of them nearly eliminated it (Dragomiretska et al. 2020). Similar effects were shown in two single‐arm studies. Both, Beer and colleagues (Beer 2016) and Pohl and colleagues (Pohl et al. 2016) examined the effect of mineral water intake in individuals experiencing a minimum of two episodes of heartburn per week over a prolonged period (≥ 3 months). In both studies, participants ingested 1500 mL of bicarbonate‐rich mineral water daily for a duration of 6 weeks. The water was predominantly consumed either before or during mealtime. None of these studies included a control group for comparison. Based on entries recorded in patients' diaries, mineral water intake led to a reduction both in frequency (89.6% and 81.0% of participants) and duration of symptoms (79.2% and 89.0% of participants). Mean numbers of episodes per week decreased by 5.1 ± 4.8 points and 4.8 ± 8.2 points. Moreover, all dimensions in the Reflux Disease Questionnaire, a questionnaire recording the symptoms of the upper gastrointestinal tract, significantly decreased in both studies. Thus, symptoms of heartburn, regurgitation, GERD and dyspepsia were significantly reduced. These improvements were paralleled by an increase in quality of life, measured in disease‐specific questionnaires (Quality of Life in Reflux and Dyspepsia, Gastrointestinal Quality of Life Index) and a health‐related questionnaire (Short‐Form 12). In line with these results, a recent study was able to demonstrate the superiority of bicarbonate‐rich mineral water over placebo in a Phase‐III study. Mineral water consumption and duration of the study were the same as in the aforementioned studies. In 84.7% of the verum group, mineral water consumption led to a reduction in the Reflux Disease Questionnaire score of ≥ 5 points. In comparison, the responder rate of the placebo group was 63.5%. Thus, the placebo‐corrected treatment effect exceeded 20%. Additionally, mineral water consumption positively influenced disease‐specific quality of life, demonstrating significant superiority over the placebo in reducing problems related to dietary intake, emotional distress, and vitality. However, sleep disturbances and physical or social functioning were not affected differently in both intervention groups (Labenz et al. 2023).

###### Mechanisms

Heartburn etiology varies across different disease presentations, including functional heartburn, reflux hypersensitivity, and GERD with or without endoscopically visible lesions. Primarily induced by acidic esophageal reflux, heartburn is commonly associated with GERD and nonerosive reflux disease (NERD). However, additional factors such as nonacidic gastroesophageal reflux (coupled with visceral hypersensitivity) and/or motor abnormalities may also contribute to symptom manifestation (Fass et al. 2020; Pohl et al. 2016; Savarino et al. 2022). As a buffer base, bicarbonate can effectively bind protons in the gastrointestinal tract, thereby neutralizing acids (Beer 2016). As already outlined in the mechanisms part of dyspeptic symptoms, its acid‐neutralizing action in the stomach results in a noteworthy elevation of the gastric pH (Grassi et al. 1993), consequently mitigating discomfort associated with increased stomach acidity and acid reflux.

#### 4.2.2 Diseases of the Lower Gastrointestinal Tract

FGIDs of the lower gastrointestinal tract show the highest prevalence of gastrointestinal disorders, with functional constipation and irritable bowel syndrome being the leading disorders (Sperber et al. 2021). Conventional treatment encompasses pain reduction and improvement in stool consistency using individual nutritional approaches and several different drugs (i.e., antidepressants, spasmolytics, laxatives) (Andresen et al. 2022; Layer 2023). Even complementary and naturopathic medical approaches (i.e., psyllium fiber therapy) may convey additional benefits (Andresen et al. 2022; Layer 2021).

##### 4.2.2.1 Functional Constipation and Irritable Bowel Syndrome

In the past decades, the effect of bicarbonate‐rich mineral water on functional constipation and irritable bowel syndrome has been evaluated in a few water studies. In these studies, mineral water consumption ranged from 500 mL/day up to 2000 mL/day and consumption periods varied from 10 days up to 6 weeks. The results indicate beneficial effects on gastrointestinal transit time (Gasbarrini 2006), bowel movements (Bothe et al. 2017; Corradini 2012; Dore et al. 2021), and gastrointestinal symptoms such as bloating (Bothe et al. 2017; Gasbarrini 2006). What at first glance appears to be a success story for high bicarbonate mineral water is actually more of a coincidence in the mineral composition of the water. The consistently high sulfate content and the high magnesium content across some of the studied mineral waters suggest these minerals are more likely responsible for the observed effects. Given the known osmotic properties of sulfate (Böhmer et al. 1999; Costantino et al. 2020), it is more plausible that the observed therapeutic effects are primarily attributable to the sulfate and magnesium content rather than to its bicarbonate content. Several studies on sulfate‐rich mineral waters have confirmed the laxative effect, regardless of the bicarbonate content (Bothe et al. 2017; Corradini 2012; Dupont et al. 2014, 2019; Naumann et al. 2016).

### 4.3 Effects of Bicarbonate‐Rich Mineral Water on Liver Function

MASLD, the most prevalent chronic liver disease, is characterized by hepatic steatosis in ≥ 5% of hepatocytes (Guo et al. 2022), in the absence of secondary causes of hepatic fat accumulation, such as excessive alcohol consumption, the use of steatogenic medications, or hereditary disorders (Chalasani et al. 2012). One of the key pathogenic mechanisms in NAFLD is insulin resistance, which leads to a significant increase in plasma free fatty acids. This overloads the fatty acid oxidation capacity in hepatocytes, resulting in mitochondrial damage, the formation of lipotoxic lipids, and endoplasmic reticulum stress. These processes trigger inflammatory responses, which, in turn, promote a broader range of inflammatory reactions and contribute to the progression of liver fibrosis (Guo et al. 2022). Additionally, individuals suffering from NAFLD frequently show imbalances in the antioxidant defense system and increased lipid peroxidation (Dragomiretska et al. 2023).

Currently, there are no clinically approved pharmacological treatments for MASLD. As a result, treatment guidelines focus primarily on dietary and lifestyle modifications (Roeb and Geier 2019). According to the German guideline for the diagnosis and treatment of MASLD, a Mediterranean diet is recommended to improve steatosis and insulin sensitivity. For overweight or obese patients, a weight loss program combined with a low‐calorie diet is advised. In contrast, for patients with normal weight, physical activity aimed at increasing muscle mass is recommended (Tacke et al. 2022). The use of bicarbonate has not been included in current treatment recommendations.

**FIGURE 5:**
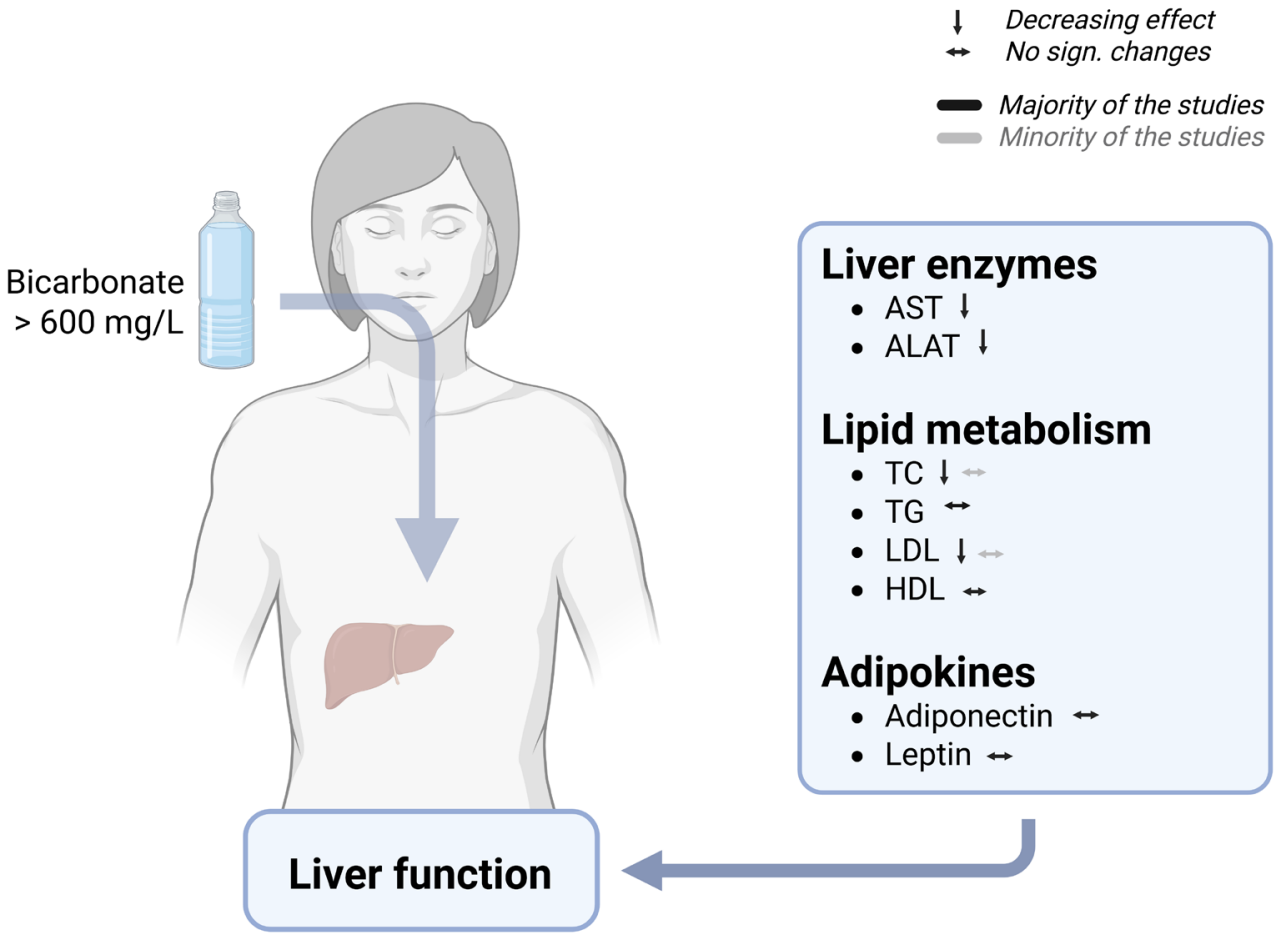
Effects of bicarbonate‐rich mineral water on the functional state of the liver. Created with BioRender. ALAT, alanine aminotransferase; AST, aspartate aminotransferase; HDL, high‐density lipoprotein; LDL, low‐density lipoprotein; MDA, malondialdehyde; TC, total cholesterol; TG, triglycerides.

There are only very few references on the effect of bicarbonate‐rich mineral water on liver function, showing beneficial effects in some aspects (Figure 5, Table 5).

**TABLE 5:** Effects of bicarbonate‐rich mineral water on liver function.

Author	Design population	Intervention	Characteristics of mineral water/treatment Bicarbonate/day	Main results	
				Time effects^a^ (Bicarbonate group)	Group differences time × water interaction
**Long‐term studies**					
Dragomiretska et al. (2020)	Parallel‐group, randomized 90 individuals with GERD	21–24 days Basic therapy: nutrition + proton pump inhibitors Basic therapy vs. basic therapy +2 different mineral water brands (water A: “Polyana Kvasova,” water B: “Donat‐Mg”) 1% of body weight (three doses, before the meals; 450–690 mL/d)	Water A: HCO_3_, Na, B Water B: HCO_3_, Mg, Na, SO_4_ HCO_3_ Both waters individualized	**Blood** (not specified)	
				Water A: n.s. time effects for functional state of the liver Water B: **TC**: ↓ **LDL**: ↓ **HDL**: ↔ **TG**: ↔ **Coefficient of atherogenicity**: ↔ **Normalization of enzymes**: ALAT ↓ AST ↓ GGT ↔ (tendency ↓)^b^ AP ↔ (tendency ↓)^b^ **Bilirubin**: ↓	Not reported
Dragomiretska et al. (2023)	Parallel‐group, randomized 71 individuals with chronic viral hepatitis C and NAFLD, after Covid‐19 infection	2 months Diet + exercise (control) vs. diet + exercise + mineral water (“Shayanskaya”) 3 mL water/kg body weight, before and after meals (3 meals)	Water: HCO_3_, Na, Si HCO_3_ Individualized	**Blood** (not specified)	
				**Lipid metabolism**: TC ↓, TG ↓, LDL ↓, HDL ↑, coefficient of atherogenicity ↓ **Carbohydrate metabolism**: glucose ↓, insulin ↓, HOMA‐IR ↓ **Adipokines**: adiponectin ↑, leptin ↓ **Liver enzymes**: ALAT ↓, AST ↓	**Lipid metabolism**: sign. group differences (LDL, coefficient of atherogenicity) **Carbohydrate metabolism:** sign. group differences (insulin, HOMA‐IR) **Adipokines**: n.s. group differences **Liver enzymes**: sign. group differences (ALAT, AST)

*Note:* Minerals: Ca, calcium; Cl, chloride; HCO_3_, bicarbonate; Mg, magnesium; Na, sodium; SO_4_, sulfate. Blood parameters: ALAT, alanine aminotransferase; AP, alkaline phosphatase; AST, aspartate aminotransferase; GGT, gamma glutamyltransferase; HOMA‐IR, homeostasis model assessment—insulin resistance; LDL, low‐density lipoprotein; TC, total cholesterol; TG, triglycerides. ↑ = significant increase (*p* < 0.05); ↔ = no significant change (*p* > 0.05); ↓ = significant decrease (*p* < 0.05).

^a^ Between begin and end of each intervention period.

^b^ Categorization of the authors.

A recent animal study demonstrated a beneficial effect of a calcium‐sulfate‐bicarbonate mineral water on MASLD: mineral water treatment improved inflammation and fibrosis via modulation of the gut‐liver axis in mice (Carpino et al. 2022). Conversely, in individuals suffering from GERD the consumption of a sodium‐ and bicarbonate‐rich mineral water over a period of 3 weeks did not alter lipid parameters and parameters of the functional state of the liver. Nevertheless, the consumption of a mineral water very rich in bicarbonate, magnesium and sulfate led to an improvement in liver enzymes (ASAT and ALT) and total cholesterol, as indicated by the second study arm in the same trial (Dragomiretska et al. 2020). To the best of our knowledge, there is only one intervention study that has evaluated the effects in humans with diagnosed MASLD. The present study comprised individuals suffering from chronic viral hepatitis C in concomitant with MASLD. All individuals had previously been diagnosed with Covid‐19, 1–2 months before the start of the study. After 2 months of consuming bicarbonate‐rich mineral water, along with a diet and exercise intervention, several indices of lipid and glucose metabolism were improved compared to the control group that did not receive mineral water. In addition, leptin and adiponectin levels were improved and the enzyme spectrum of the liver was normalized (Dragomiretska et al. 2023). In the initial human study (Dragomiretska et al. 2020), the effects appear to originate more from the sulfate. This conclusion is based on the fact that both mineral waters were rich in bicarbonate (4500–800 mg/L and 7700 mg/L, respectively), but only one of the waters contained significant amounts of sulfate (25 mg/L and 2400 mg/L, respectively). However, the observed improvements in the functional state of the liver were exclusively attributable to the sulfate‐rich mineral water. Consequently, the role of bicarbonate appears to have been negligible in this study. In contrast, the second study reported a significantly lower sulfate content of 43 mg/L (Dragomiretska et al. 2023), suggesting that the observed effects may be attributable to the elevated bicarbonate levels. However, the daily bicarbonate intake in the latter study was likely lower than in the first study. Precise statements regarding this matter are unattainable due to the utilization of individualized drinking amounts in both studies and the incomplete reporting of mineral water ingredients in one of the publications. In summary, no clear hepatoprotective effect can be derived from these two intervention studies. However, the consumption of a mineral water rich in sulfate (1590 mg/L) but low in bicarbonate (266 mg/L) over a period of 6 months showed no changes in lipid metabolism parameters and liver hormones (Gravina et al. 2022), which suggests no effects from a high sulfate content. Therefore, the influence of bicarbonate remains to be clarified.

#### Mechanisms

Since insulin resistance is a key contributor to the development of MASLD, improving the functional state of the liver appears to be closely linked to the reduction in insulin resistance. For a detailed discussion of the proposed mechanisms underlying the improvement in insulin resistance, please refer to the section titled “Effects of bicarbonate‐rich mineral water on glucose metabolism.”

## 5 Limitations of the Trials

The studies included in this review present several methodological limitations that must be taken into account when interpreting the overall findings. With the exception of studies on gastrointestinal complaints and liver health, most trials had relatively small sample sizes, typically involving around 20 participants, which limits both statistical power and generalizability. Only a small subset of studies addressing cardiovascular risk factors (*n* = 5) included more than 50 participants, thereby reducing confidence in the robustness of the reported effects. Study design quality represented another notable limitation. Randomization was inconsistently implemented, and reporting on blinding was often inadequate. Only three studies explicitly reported using a double‐blind design, and four employed single‐blinding. In contrast, three studies clearly stated that no blinding was applied, while 18 did not mention blinding procedures at all. However, three of these were single‐arm studies, for which blinding is typically not applicable. The frequent lack of blinding across the reviewed studies introduces a considerable risk of performance and detection bias. Regarding study design types, more than one‐third of the included papers used a parallel‐group design rather than a crossover design (9 parallel vs. 13 crossover). Although both designs have their respective advantages, crossover trials generally provide greater statistical efficiency for within‐subject comparisons—an important consideration in studies with small sample sizes. Thus, the limited use of crossover designs may represent a missed opportunity to enhance internal validity and reduce interindividual variability. Furthermore, studies on upper gastrointestinal complaints frequently lacked a control group (five out of nine studies), limiting the ability to draw causal inferences. This absence hinders the differentiation between true intervention effects and changes due to confounding factors or natural symptom variability. Additionally, the null findings related to blood glucose levels should be interpreted with caution. As many of the studies included healthy participants, clinically meaningful changes in glycemic parameters were unlikely to occur. To adequately evaluate the potential health benefits of mineral water consumption, future studies should include individuals with an adverse metabolic profile, such as those with type 2 diabetes or impaired glucose tolerance. Moreover, effects should be tested in individuals without glucose‐lowering medication.

It should be noted that the effects described cannot be attributed exclusively to the presence of bicarbonate. As stated in the part “mineral water with a high bicarbonate content,” the bicarbonate‐rich mineral waters utilized in the summarized studies occasionally contain varying levels of other minerals, including potassium, magnesium, sodium, and calcium. These minerals may have contributed to the outcomes observed in the respective studies. However, the present review deliberately focused on mineral water, in which bicarbonate was the predominant biochemical constituent. Comprehensive reviews on different types of mineral water are given elsewhere (Naumann et al. 2017; Costa‐Vieira et al. 2019; Sokrateva et al. 2025). In addition, the literature provides evidence that fluid intake itself influences cardiometabolic factors (Brunkwall et al. 2020; Koceva et al. 2025; Vanhaecke et al. 2021). This association has often been ignored in the studies summarized here, which may have affected the interpretation of the results. A possible increase in fluid intake due to the study conditions could have contributed to the positive findings observed in some control groups.

In light of these limitations, future research in this field should prioritize rigorous methodological standards. Well‐powered, randomized, double‐blind, controlled trials employing a crossover design are needed, particularly in populations with diagnosed metabolic disorders such as type 2 diabetes or hypercholesterolemia. Adequate sample sizes are essential to improve statistical power, enhance generalizability, and increase the external validity of findings. Finally, dietary assessment should be systematically integrated into study protocols—ideally through the implementation of a controlled diet; at a minimum, validated tools such as food frequency questionnaires or 3‐day dietary records should be employed to account for potential dietary confounders.

## 6 Summary

The regulation of systemic acid–base balance represents a fundamental aspect of human physiology, increasingly recognized as being modifiable through dietary choices. Modern Western dietary patterns, typically rich in acidifying animal‐based foods such as meat and cheese, contribute to a persistently elevated dietary acid load, which has been linked to adverse metabolic and cardiovascular outcomes. Bicarbonate‐rich mineral waters offer a physiologically relevant means to counteract diet‐induced subchronic metabolic acidosis and have been associated with beneficial effects across multiple organ systems, including cardiovascular health, gastrointestinal function, and liver metabolism.

From a mechanistic perspective, the alkalinizing effect of bicarbonate‐rich mineral water may improve insulin sensitivity by enhancing insulin receptor binding—an effect supported by epidemiological data linking higher bicarbonate levels to lower insulin resistance, demonstrated by lower fasting insulin concentrations. An additional mechanism potentially involved includes the modulation of lactate dynamics. While glucocorticoid involvement in insulin resistance is plausible under acidic conditions, current data do not support a consistent impact of bicarbonate‐rich mineral water on cortisol levels. Regarding lipid metabolism, both osmotic and pH‐dependent effects have been proposed to explain reductions in cholesterol and postprandial triglycerides. Mechanisms include reduced cholesterol absorption due to altered intestinal conditions and increased fecal bile acid excretion, possibly driven by changes in gallbladder motility and bile flow. However, variations in mineral content and dosage complicate the interpretation of these findings. Blood pressure effects through bicarbonate‐rich mineral water appear to involve the renin–angiotensin–aldosterone system. While acute studies indicate a downregulation of aldosterone secretion, long‐term effects are less consistent and may depend on sodium content and the amount of bicarbonate consumed.

From the clinical perspective, the consumption of bicarbonate‐rich mineral water shows promising prospects for long‐term improvements in glycemic control and cholesterol levels, with potential positive implications for cardiovascular health. Despite the high sodium content present in many of these mineral waters, the effects on blood pressure are generally neutral to positive. Moreover, the consumption of bicarbonate‐rich mineral water has demonstrated symptom‐relieving effects in individuals with upper gastrointestinal complaints, such as dyspepsia and heartburn. These benefits likely result from both acid‐buffering capacity and improved gastric motor function, possibly mediated by elevated gastrin secretion. Importantly, cooccurring minerals, particularly calcium, may further support esophageal acid clearance and peristalsis.

Taken together, the evidence suggests that bicarbonate‐rich mineral water may serve as a nonpharmacological approach to mitigate acid load and improve parameters of metabolic, cardiovascular, and gastrointestinal health. However, variations in study design, population characteristics, and water composition underscore the need for further research.

## Conflicts of Interest

The authors declare no conflicts of interest.

## Supporting information


**Figure S1:** Effects of bicarbonate‐rich mineral water on glucose metabolism (referred to the baseline status). Created with BioRender.
**Figure S2:** Effects of bicarbonate‐rich mineral water on lipid metabolism (referred to the baseline status). Created with BioRender.

## Data Availability

The data that support the findings of this study are available from the corresponding author upon reasonable request.
