# Supplementary material for: Beyond Thirst: Influence of Bicarbonate Mineral Water on Cardiovascular Risk Factors, Gastrointestinal Function, and Liver Health
Source: Food Sci Nutr. 2025 Dec 9;13(12):e71183. doi: 10.1002/fsn3.71183 (PMC12687301; doi:10.1002/fsn3.71183)
Supplement: Supplementary file 1 — Figure S1: Effects of bicarbonate‐rich mineral water on glucose metabolism (referred to the baseline status). Created with BioRender. Figure S2: Effects of bicarbonate‐rich mineral water on lipid metabolism (referred to the baseline status). Created with BioRender. [file FSN3-13-e71183-s001.docx]

**Beyond Thirst: Influence of Bicarbonate Mineral Water on Cardiovascular Risk Factors, Gastrointestinal Function and Liver Health**

**Author information**

Katharina Mansouri^1^, Maximilian Andreas Storz^2^, Thierry Hanh^3^, Andreas Hahn^1^

^1^*Institute of Food and One Health, Leibniz University Hannover, Am Kleinen Felde 30, 30159 Hanover, Germany*

^2^ *Department of Internal Medicine II, Center for Complementary Medicine, Medical Center - University of Freiburg, Faculty of Medicine, University of Freiburg, Freiburg, Germany*

^3^*Independent Researcher, Paris, France*


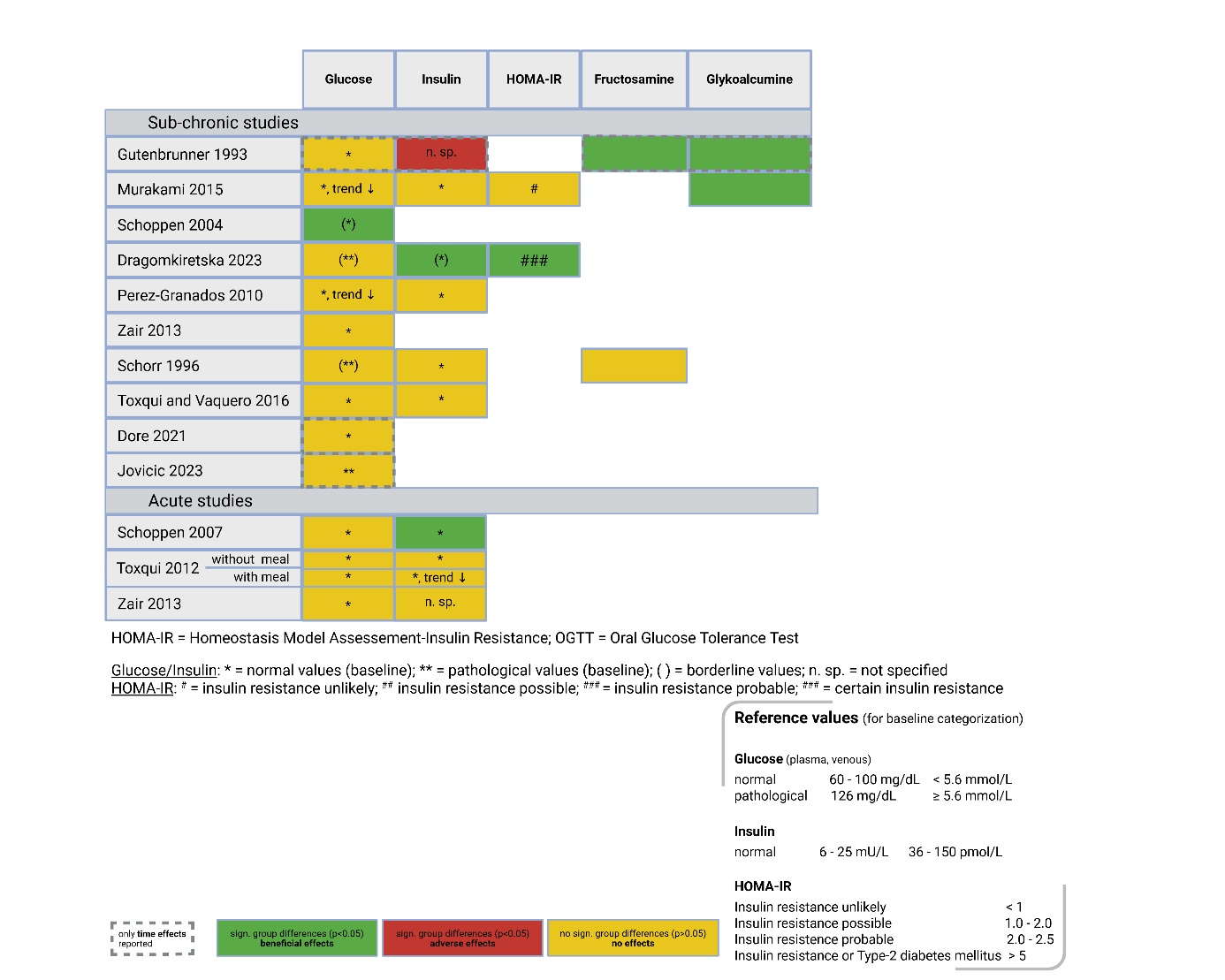


*Figure S1*: Effects of bicarbonate-rich mineral water on glucose metabolism (referred to the baseline status). Created with BioRender.


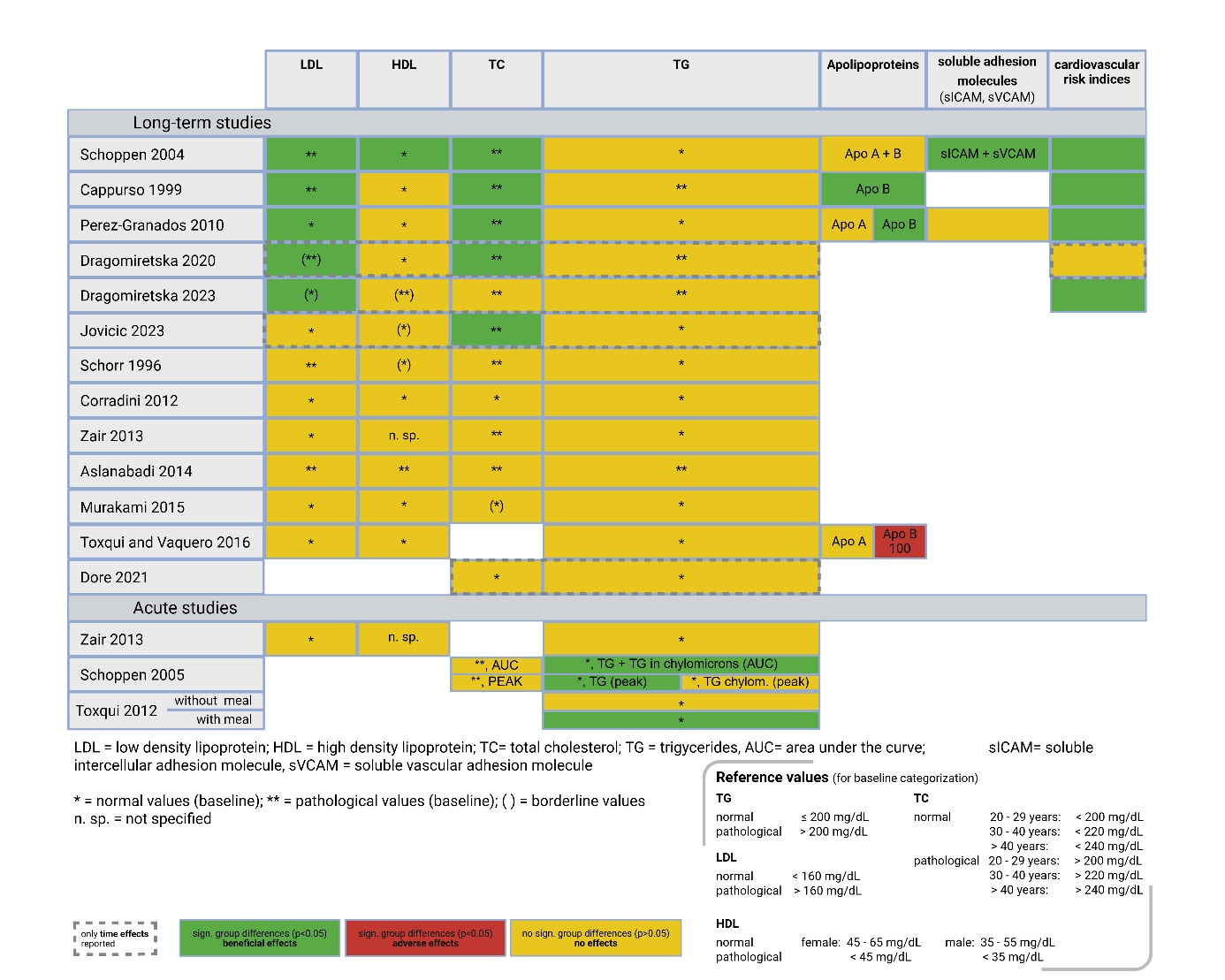


*Figure S2*: Effects of bicarbonate-rich mineral water on lipid metabolism (referred to the baseline status). Created with BioRender.
